# Dynamics of the Bacillus subtilis Min System

**DOI:** 10.1128/mBio.00296-21

**Published:** 2021-04-13

**Authors:** Helge Feddersen, Laeschkir Würthner, Erwin Frey, Marc Bramkamp

**Affiliations:** a Institute for General Microbiology, Christian-Albrechts-University Kiel, Kiel, Germany; b Arnold-Sommerfeld-Center for Theoretical Physics and Center for NanoScience, Ludwig-Maximilians-Universität München, Munich, Germany; c Central Imaging Facility, Christian-Albrechts-University Kiel, Kiel, Germany; Vanderbilt University

**Keywords:** *B. subtilis*, Min system, cell division, FRAP, PALM, super resolution microscopy, protein patterns, reaction diffusion equations

## Abstract

Division site selection is a vital process to ensure generation of viable offspring. In many rod-shaped bacteria, a dynamic protein system, termed the Min system, acts as a central regulator of division site placement. The Min system is best studied in Escherichia coli, where it shows a remarkable oscillation from pole to pole with a time-averaged density minimum at midcell. Several components of the Min system are conserved in the Gram-positive model organism Bacillus subtilis. However, in B. subtilis, it is commonly believed that the system forms a stationary bipolar gradient from the cell poles to midcell. Here, we show that the Min system of B. subtilis localizes dynamically to active sites of division, often organized in clusters. We provide physical modeling using measured diffusion constants that describe the observed enrichment of the Min system at the septum. Mathematical modeling suggests that the observed localization pattern of Min proteins corresponds to a dynamic equilibrium state. Our data provide evidence for the importance of ongoing septation for the Min dynamics, consistent with a major role of the Min system in controlling active division sites but not cell pole areas.

## Abstract (precis)

The molecular mechanisms that help to place the division septum in bacteria is of fundamental importance to ensure cell proliferation and maintenance of cell shape and size. The Min protein system, found in many rod-shaped bacteria, is thought to play a major role in division site selection.

## INTRODUCTION

The spatiotemporal regulation of cell division in bacteria is an essential mechanism ensuring correct partitioning of DNA to produce viable daughter cells upon division. The best-studied model organisms in this aspect are the rod-shaped Gram-positive and Gram-negative bacteria Bacillus subtilis and Escherichia coli, respectively. Both species divide precisely at the geometric middle via binary fission. The earliest observed event in this process is the formation of the Z-ring, a ring-like structure consisting of polymerized FtsZ proteins, a highly conserved homologue of eukaryotic tubulin (1–5). Once assembled, FtsZ acts as a dynamic scaffold and recruits a diverse set of proteins forming the divisome, a complex that mediates cytokinesis (6–8). Recently, treadmilling of FtsZ filaments was shown to drive circumferential peptidoglycan (PG) synthesis (9–11). However, it is still not fully understood how FtsZ finds the precise midplane of the cell. In E. coli and B. subtilis, the nucleoid occlusion (NO) and the Min system, two negative FtsZ regulators, have been shown to confine its action spatially to the center of the cell. Noc in B. subtilis and SlmA in E. coli bind to DNA and inhibit FtsZ polymerization across the nucleoid (12–16).

The Min system in E. coli consists of the three proteins MinC, MinD, and MinE (17, 18) and has been studied extensively both experimentally (19–31) and theoretically (31–37). MinC is the inhibitor of Z-ring formation, inhibiting the bundling of FtsZ filaments (24, 38–41). MinC is localized through MinD, a protein that belongs to the WACA (Walker A cytomotive ATPase) family (42, 43). Upon binding ATP, MinD dimerizes and associates with the membrane through a conserved C-terminal membrane targeting sequence (MTS) (3, 44, 45). MinC and MinD have been described to form large ATP-dependent alternating polymers that assemble cooperatively and locally inhibit FtsZ bundling (46, 47). In the absence of MinCD, cells frequently produce the name-giving anucleate minicells (48, 49). The E. coli MinCD complexes are disassembled and detached from the membrane by MinE, a protein that forms a ring-like density profile at the rim of MinD assemblies (50, 51) and binds to the membrane via an amphipathic helix serving as MTS (52, 53). MinE triggers ATPase activity of MinD, leading to membrane detachment of MinCD (29). Cytosolic MinD rebinds ATP and binds the membrane again, thereby leading to a remarkably robust oscillation of the Min system in E. coli (27, 29, 54, 55). Min protein dynamics are a paradigmatic example of cellular self-organization (56). Due to the simplicity of the system, it has been subject to several molecular modeling studies and *in vitro* reconstructions (28–37).

The Min system in B. subtilis lacks MinE as the essential factor that is responsible for Min oscillation in E. coli, and therefore the Min proteins do not oscillate in B. subtilis. Even though the original publications only vaguely suggest this (57, 58), the B. subtilis Min proteins are often described to form a stationary bipolar gradient decreasing toward midcell (3, 8), therefore restricting assembly of a functional FtsZ ring to the midplane of the cell. The spatial cue for a gradient in B. subtilis is provided by DivIVA (59, 60). DivIVA targets and localizes to negatively curved membrane regions (61). MinJ acts as a molecular bridge between MinD and DivIVA (62, 63). MinJ contains six predicted transmembrane helices and a PDZ domain, which often participate in protein-protein interactions (64). Due to the polar targeting of DivIVA, MinCDJ should form a stationary polar gradient decreasing toward midcell, restricting FtsZ polymerization spatially (57, 58). However, several studies suggest that the B. subtilis Min system may rather act downstream of FtsZ ring formation by preventing reinitiation of division at former sites of cytokinesis (62, 63, 65), including some of the very early work (58).

We have recently analyzed DivIVA dynamics in B. subtilis and found that Min proteins redistribute from the cell poles to midcell as soon as a septum is formed (66), which prompted us to reanalyze Min protein dynamics in this organism. To this end, we generated a set of new fusions to DivIVA, MinD and MinJ. To avoid overexpression artifacts that would corrupt protein dynamics studies, we generated strains where the native gene copies were replaced by functional fluorescent fusions. These allelic replacements were used to determine precise molecule counts per cell. Using fluorescent recovery after photobleaching (FRAP), we determined the protein dynamics of the individual Min proteins. We then calculated protein diffusion coefficients that were further used for modeling and simulations of the observed Min dynamics. We finally analyzed the nanoscale spatial distribution of the Min proteins in B. subtilis by single-molecule localization microscopy (SMLM). Our data are consistent with a dynamic turnover of MinD between membrane and cytosol. Moreover, our SMLM data support a model in which the Min complex is in a dynamic steady state that is able to relocalize from the cell pole to the septum facilitated by a geometric cue, namely, the invagination of the membrane at the septum. Based on our experimental data, we propose a minimal theoretical model for the Min dynamics in B. subtilis in realistic three-dimensional (3D) cell geometry. The model is based on a reaction-diffusion system for MinD and incorporates the effects of DivIVA and MinJ implicitly through space-dependent recruitment and detachment processes. Our computational analysis of the mathematical model reproduces qualitative features of the Min dynamics in B. subtilis and shows that localization of MinD to the poles or septum corresponds to a dynamic equilibrium state. Furthermore, our model suggests that a geometric effect alone could explain septum localization of MinD once DivIVA is recruited to the growing septum, therefore highlighting the importance of geometry effects that cannot be captured in a simplified one-dimensional (1D) model.

## RESULTS

### Construction of fluorescent fusions with native expression level.

Even though the Min system in B. subtilis has been extensively investigated before, most studies were conducted using strains that overexpress fluorescent fusions from ectopic locations upon artificial induction (57, 58), leading to nonnative expression levels that can alter the native behavior of fine-tuned systems like the Min system. Additionally, even small populations of a protein from overexpression make it difficult to identify a dynamic fraction through diffraction-limited microscopy (67). Hence, we aimed to recharacterize the dynamics of the Min components in B. subtilis by using strains that avoid or minimize overexpression artifacts and, hence, created a set of allelic replacements (see Fig. S1a in the supplemental material).

10.1128/mBio.00296-21.2 FIG S1 Cartoon of strain construction strategy for allelic replacement in B. subtilis. (a) All strains in this study were created to express fluorophore fusions from their native promoter to sustain native protein levels. Furthermore, they were tested for functionality. Construction of plasmids was performed with Golden Gate cloning, yielding a plasmid that can be directly transformed into B. subtilis. After transformation, genes for a fluorophore and an antibiotic resistance cassette were integrated into the genomic locus of interest via homologous recombination. (b) Microscopic images of a selection of strains used in this study. Columns from left to right: phase contrast, red fluorescent channel using membrane dye (FM4-64), green fluorescent channel depicting the indicated fluorophore, and composite of all three channels. Scale bars, 2μm. Download FIG S1, TIF file, 2.5 MB. Copyright © 2021 Feddersen et al. 2021 Feddersen et al. https://creativecommons.org/licenses/by/4.0/ This content is distributed under the terms of the Creative Commons Attribution 4.0 International license.

Dysfunctionality or deletion of Min components in B. subtilis manifests in an easily observable phenotype of increased cell length and DNA-free minicells (Table 1). This allows rapid evaluation of the functionality of fluorescent fusions in the constructed strains by comparing cell length and number of minicells between mutant and wild-type strains (Table 1).

**TABLE 1:** Phenotypic characterization of relevant strains^*a*^

Strain	Description of strain	Mean growth rate constant (μ) ± SD	Mean cell length (μm) ± SD	% Minicells
168	Wild type	0.53 ± 0.004	3.11 ± 0.77	0.3
3309	Δ*minCD*	0.45 ± 0.021	7.64 ± 2.70	45.8
RD021	Δ*minJ*	0.51 ± 0.049	6.65 ± 2.02	13.8
4041	Δ*divIVA*	0.46 ± 0.020	8.13 ± 3.40	29.6
BHF011	Dendra2-MinD	0.49 ± 0.004	2.67 ± 0.61	0.9
BHF017	msfGFP-MinD	0.55 ± 0.004	4.22 ± 1.04	9.1
JB38	MinJ-Dendra2	0.51 ± 0.006	3.44 ± 1.06	0
BHF007	MinJ-msfGFP	0.57 ± 0.013	3.38 ± 0.76	0.3
JB40	MinJ-mNeonGreen	0.57 ± 0.002	3.16 ± 0.67	0
JB36	DivIVA-Dendra2	0.50 ± 0.007	4.33 ± 0.92	8.0
1803	DivIVA-GFP	0.45 ± 0.021	3.31 ± 0.73	1.1
BHF028	DivIVA-mNeonGreen	0.54 ± 0.029	5.42 ± 1.35	5.3
JB37	DivIVA-PAmCherry	0.51 ± 0.019	4.35 ± 1.11	3.3

a For determination of the growth rate constant, μ, the optical density at 600 nm of exponentially growing cells was measured. Cell length and the percentage of minicells were determined microscopically using Fiji, with *n* ≥ 200. Strains were grown in independent triplicates, with differences reflected in the standard deviation (SD).

Here, we generated functional fusions to MinD (Dendra2 [68]) and MinJ (monomeric superfolder GFP [msfGFP] [69] and mNeonGreen [70]), as judged by cell length, number of minicells, and subcellular protein localization (Fig. S1b; Table 1). Dendra2-MinD displayed a phenotype comparable to that of the wild type. Unfortunately, Dendra2-MinD could not be used for FRAP studies, because excitation at 488 nm leads to a significant green-to-red conversion during the course of the experiment. When all proteins were converted from green to red prior to the FRAP experiment with UV light (405 nm), the red fluorescent signal was poor and bleaching of most proteins occurred during the first image acquisitions, prohibiting reliable quantification. Upon converting protein locally at one of the poles or a septum with a short laser pulse at 405 nm and subsequent imaging in the red channel, very fast diffusion of converted Dendra2-MinD throughout the cell could be observed (data not shown). However, the signal was too dim to be quantified satisfactorily.

Therefore, another strain expressing msfGFP fused to MinD was created. This fusion protein was at least partially functional according to cell length and number of minicells (Fig. S1b; Table 1). When *msfGFP-minD* was transformed in a genetic background of a Δ*minJ* or Δ*divIVA* mutant, the fluorescent signal was, as expected, distributed in the cytosol, sometimes forming small foci. MinJ-msfGFP also lost its polar and septal localization upon deletion of *divIVA*, as reported previously (62). These strains were not used for further analysis of protein dynamics, because without protein interaction, a merely diffusive behavior will dominate and no further insight into Min protein dynamics and interaction will be gained. We also aimed at constructing membrane-binding mutants in which the MTS of MinD was altered. However, we were not able to create viable strains with allelic replacement of the native *minD* gene.

When DivIVA fluorescent fusions were constructed, several different fluorophores (FPs) were successfully fused to DivIVA, namely, mCherry2, mNeonGreen, Dendra2, PAmCherry, mGeosM, and Dronpa (68, 70–74), with linkers of between 2 and 15 amino acids. Unfortunately, all of them showed a mild or strong phenotype, some even severe protein mislocalization, hinting toward limited functionality of these DivIVA fusion proteins (75; and data not shown). Since this did not meet the set standards for this study, we turned toward strain 1803 (76), carrying a *divIVA-GFP* copy with its native promoter in the ectopic *amyE* locus. While DivIVA-green fluorescent protein (GFP) has been shown to not fully complement a Δ*divIVA* strain (76, 77), it still localizes correctly and can be used for studies of DivIVA dynamics (66, 77). Additionally, we performed FRAP on DivIVA-mNeonGreen, which shows only a mild phenotype (Table 1), in wild-type and Min knockout backgrounds to be able to compare it with the effect of the extra copy of DivIVA in strain 1803 (Fig. S2).

10.1128/mBio.00296-21.3 FIG S2 Representative microscopy images of FRAP analysis of DivIVA-mNeonGreen and DivIVAd34-mNG. (a) DivIVA-mNeonGreen expressed in wild-type background (BHF028) and DivIVAd34-mNG (BHF067). Images were taken before bleaching of the indicated spot with a 488-nm laser pulse, directly after bleaching, and after recovery of fluorescence. Scale bars, 2 μm. (b) Representation of the normalized fluorescence recovery in the green channel over time. *T*_1/2_ is time when fluorescence recovery reaches half the height of total recovery, indicated on the graph with a dashed square. The red line represents measured values, the black line represents the fitted values. (c) Representative microscopy images of DivIVAΔ34-mNeonGreen localization in B. subtilis. To investigate the localization of DivIVAΔ34-mNeonGreen, cells of B. subtilis strain BHF067 were grown in MD medium to an OD_600_ of 0.5 and loaded onto a 1.5% agarose pad in MD medium. Upper and lower panels show different cells expressing DivIVAΔ34-mNeonGreen. The left panels show images obtained by phase contrast, while the right panels show green fluorescence. Scale bar, 2 μm. Download FIG S2, file, MB. Copyright © 2021 Feddersen et al. 2021 Feddersen et al. https://creativecommons.org/licenses/by/4.0/ This content is distributed under the terms of the Creative Commons Attribution 4.0 International license.

All fluorescent fusions were analyzed via SDS-PAGE with subsequent visualization through in-gel fluorescence or Western blotting (Fig. S3). We used in-gel fluorescence to obtain estimations about the number of molecules of the Min proteins during mid-exponential phase. We calculated protein numbers relative to the total amount of MinD that was quantified under the same growth conditions using mass spectrometry described previously (78) (Table 2; Fig. S4). MinD proteins are highly abundant (3,544 proteins per cell), while DivIVA numbers are less than 50% of that (1,690 proteins per cell). MinJ has only 16% of MinD abundancy (576 proteins per cell).

**TABLE 2:** Relative quantification of Min proteins fused to Dendra2^*a*^

Protein	Relative amount (%)	Total no. of copies/cell
MinD	100 ± 2.51	3,544 ± 89
MinJ	16.25 ± 4.36	576 ± 25
DivIVA	47.70 ± 3.51	1,690 ± 59

a Relative amounts of protein were determined via in-gel fluorescence of biological triplicates of cell lysates (see Fig. S4 in the supplemental material). Absolute protein quantities were determined relative to MinD, which was quantified in another publication (75) under similar conditions. Values are shown with standard deviations (SD).

10.1128/mBio.00296-21.4 FIG S3 Western blots or in-gel fluorescence of native Min protein fusions. To control for full-length fluorescent protein fusions with MinD, MinJ, or DivIVA, cell lysates were either fully (96°C for 10 min [e]) or partially (room temperature for 20 min [a, b, c, d, f, and g]) denatured and separated via SDS-PAGE. Protein bands were then visualized either directly via in-gel fluorescence (d, f, and g) with excitation and emission at 488/526 nm, respectively, or colorimetrically via Western blotting with the respective indicated antibody (polyclonal anti-Dendra2 [a]; monoclonal anti-mNeonGreen [b and c]; polyclonal anti-mCherry [e]). Download FIG S3, TIF file, 1.0 MB. Copyright © 2021 Feddersen et al. 2021 Feddersen et al. https://creativecommons.org/licenses/by/4.0/ This content is distributed under the terms of the Creative Commons Attribution 4.0 International license.

10.1128/mBio.00296-21.5 FIG S4 Relative quantification of native Dendra2 fusions assayed by in-gel fluorescence of SDS-PAGE gels. Biological triplicates are indicated by a number at the top right (1 to 3). Lysates of the respective strain were partially denatured with SDS loading dye at room temperature for 20 min, loaded in different relative amounts (left, 1×; right, 2×), and separated via SDS-PAGE. (a) Visualization via Typhoon Trio scanner, with excitation at 488 nm and an emission filter of 526 nm. (b) Coomassie blue stain of the respective image as a loading control. Results of quantification can be found in Table 2. Download FIG S4, TIF file, 2.3 MB. Copyright © 2021 Feddersen et al. 2021 Feddersen et al. https://creativecommons.org/licenses/by/4.0/ This content is distributed under the terms of the Creative Commons Attribution 4.0 International license.

### The Min system in B. subtilis is in a dynamic steady state.

**FIG 1:**
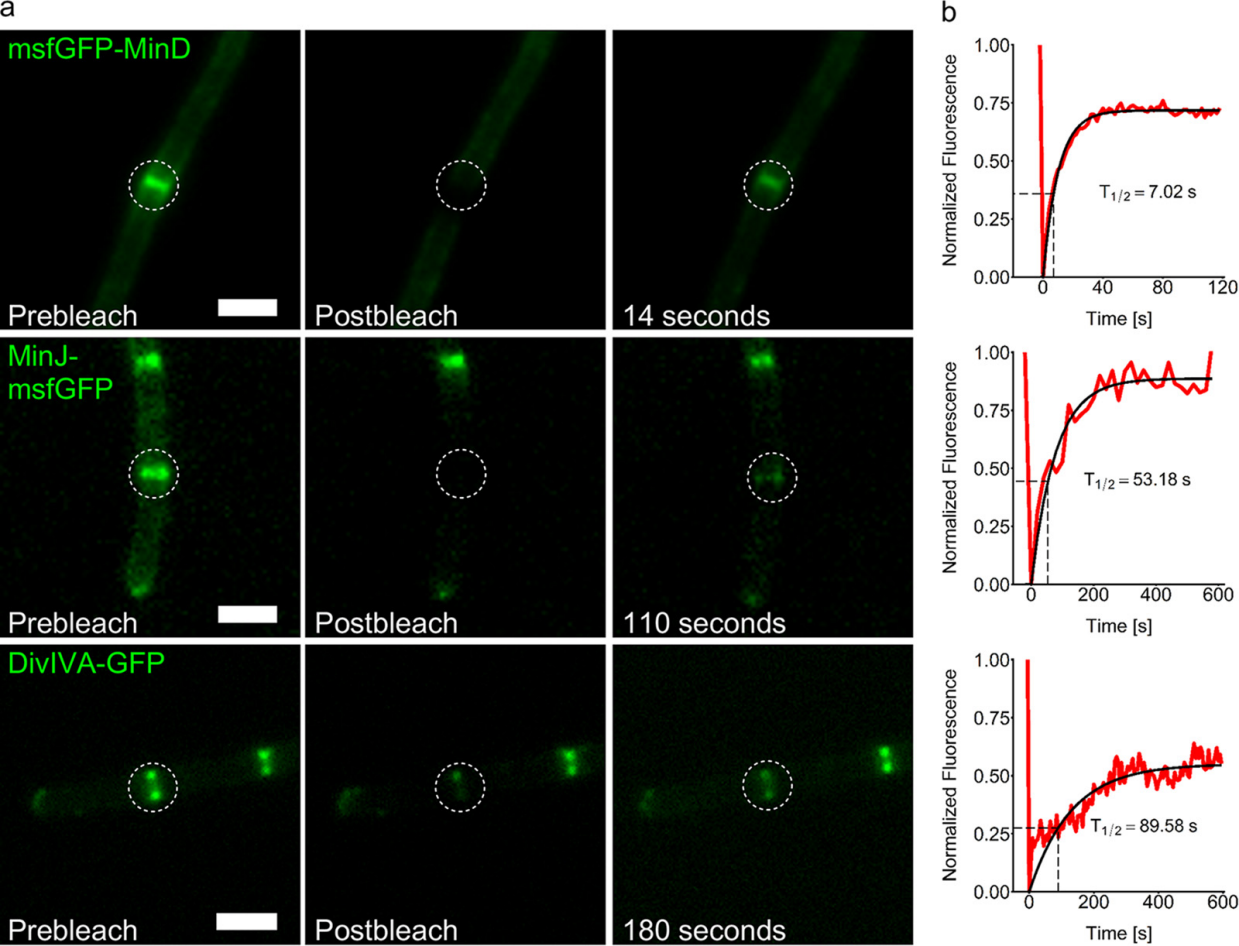
FRAP experiments in growing B. subtilis cells reveal Min protein dynamics. (a) Representative microscopy images of msfGFP-MinD (BHF017), MinJ-msfGFP (BHF007), and DivIVA-GFP (1803) before bleaching of the indicated spot with a 488-nm laser pulse, directly after bleaching, and after recovery of fluorescence. Scale bars, 2 μm. (b) Representation of the normalized fluorescence recovery in the green channel over time. *T*_1/2_ = time when fluorescence recovery reaches half height of total recovery; the shown value corresponds to the displayed cell, indicated on the graph with a dashed square. The red line represents measured values of the displayed cell, and the black line represents the fitted values. Values were obtained as described in Materials and Methods (equations 1 to 3).

**FIG 2:**
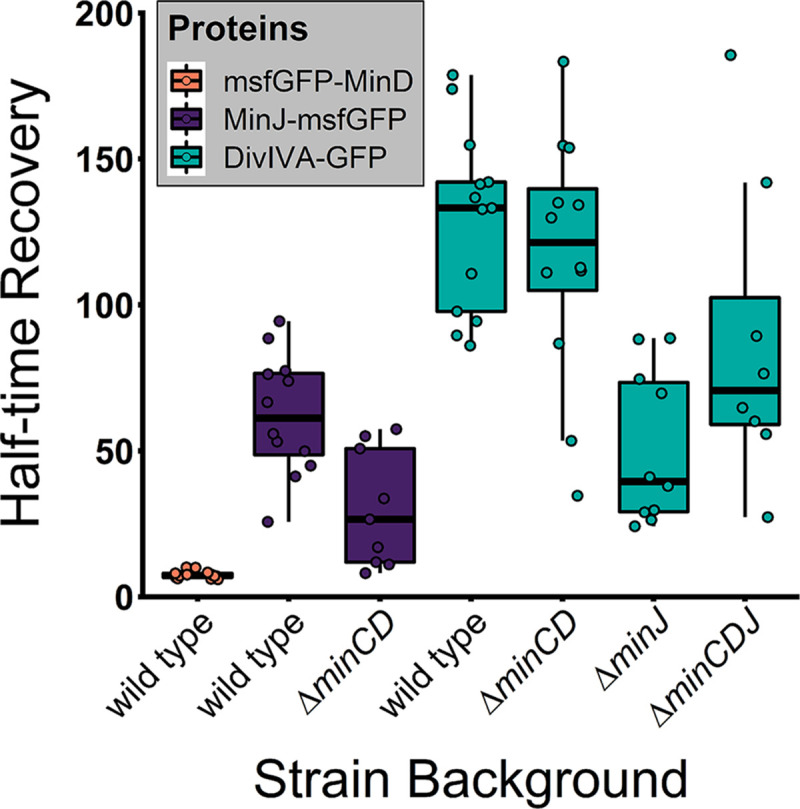
B. subtilis Min proteins form dynamic complexes. Shown are median half-time recovery values, indicated by the black bar inside each box. Each box represents a different strain; see also Table 3 for mean values. Every dot represents a single FRAP experiment (*n* ≥ 8).

Strains expressing functional Min fusions were then used for microscopic analysis of protein dynamics using fluorescent recovery after photobleaching (FRAP) experiments. All three components of the Min system showed relatively fast diffusion in FRAP (Fig. 1 and 2; Table 3). A strain expressing msfGFP-MinD (BHF017) was used for FRAP analysis of MinD dynamics. We observed a fast fluorescence recovery (time when fluorescence recovery reaches half of total recovery [*T*_1/2_] = 7.55 s), indicating rapid exchange of MinD molecules around the division septum, similar to what was previously reported for MinC (67). Bleaching of MinD at a septum was very efficient (Fig. 1a, upper panel), and the exchange of MinD molecules at the bleached spot appeared to include proteins localized distant from the bleached septum as well as in the vicinity, since the fluorescent signal in the cell decreased evenly over the whole cell length during recovery. Furthermore, around 79% of the msfGFP-MinD population appeared to be mobile (Fig. 1; Table 3). Next, we investigated MinJ-msfGFP fluorescence recovery, which was considerably slower than that of msfGFP-MinD but still indicating protein diffusion (*T*_1/2_ = 62.4 s). MinJ contains six predicted transmembrane helices, and therefore, a slower recovery was expected. Again, most of the MinJ-msfGFP protein pool appeared to participate in the fluorescence recovery (77%). When we measured DivIVA-GFP and DivIVA-mNeonGreen dynamics at septal localizations using FRAP, we observed similar mobilities (DivIVA GFP *T*_1/2_ = 128 s; DivIVA-mNeonGreen *T*_1/2_ = 60.3 s). Since the DivIVA-GFP-expressing strain has an extra copy of *divIVA*, it seems logical that the recovery time roughly doubles compared to the DivIVA-mNeonGreen-expressing strain with only one copy of the gene. DivIVA has previously been reported as static (77); however, those FRAP experiments were carried out using overexpression strains and a much shorter time frame than here. Earlier observations from our own lab using a merodiploid strain have already suggested that DivIVA is dynamic (66). Roughly two-thirds of DivIVA molecules were participating in dynamics. Since DivIVA is cytosolic while MinJ is a membrane protein, it was surprising that both proteins presented similar fluorescence recovery speeds. To test if the comparatively slow recovery of DivIVA can be explained only by its ability to oligomerize, we made use of a previously described oligomerization mutant, DivIVAΔ34 (79). Despite still being able to dimerize and bind the plasma membrane, this mutant is unable to form larger DivIVA multimers (79), and a corresponding strain expressing DivIVAΔ34-mNeonGreen was constructed (BHF067). Fluorescent imaging of this strain revealed a loss in polar and septal stabilization and localization of DivIVA (Fig. S2a to c). Instead, DivIVAΔ34-mNeonGreen was observed inhomogeneously distributed in the cytosol, with no apparent tendency for membrane binding (Fig. S2c). In FRAP experiments, recovery of DivIVAΔ34-mNeonGreen was almost instantaneous (Fig. S2a). It is, however, difficult to measure diffusion coefficients of freely diffusing proteins accurately by FRAP in bacteria, because of the small cellular volume (80). The observed result confirmed the prediction that DivIVA mobility is affected mainly by its ability to oligomerize, which not only stabilizes the protein but also affects its ability to sense negative curvature (79).

**TABLE 3:** Results of FRAP analysis for Min proteins in different genetic backgrounds

Protein and genetic background	Fluorophore	Diffusion coefficient (μm² · **10**^**−3**^** · s**^**−1**^)	Half-time recovery (s)	Mobile fraction
MinD in wild type	msfGFP	57.8 ± 10.1	7.55 ± 1.31	0.79
MinJ in wild type	msfGFP	7.19 ± 2.27	62.4 ± 19.7	0.77
MinJ in Δ*minCD* mutant	msfGFP	14.5 ± 9.54	30.2 ± 19.9	0.75
DivIVA in wild type	GFP	3.39 ± 0.82	128 ± 30.9	0.65
DivIVA in Δ*minCD* mutant	GFP	3.74 ± 1.36	116 ± 42.4	0.68
DivIVA in Δ*minJ* mutant	GFP	8.57 ± 4.43	50.9 ± 26.4	0.49
DivIVA in Δ*minCDJ* mutant	GFP	4.98 ± 2.93	87.7 ± 51.6	0.61
DivIVA in wild type	mNeonGreen	7.23 ± 1.99	60.3 ± 16.6	0.64
DivIVA in Δ*minCD* mutant	mNeonGreen	6.88 ± 2.76	63.4 ± 25.4	0.67
DivIVA in Δ*minJ* mutant	mNeonGreen	18.0 ± 3.22	24.4 ± 4.33	0.39
DivIVA in Δ*minCDJ* mutant	mNeonGreen	9.47 ± 4.26	46.1 ± 20.7	0.66

### Interaction of Min proteins influences their dynamics.

To obtain a better understanding of the interactions between Min proteins and to find an explanation for the observed dynamics, we performed FRAP experiments in various genetic knockout backgrounds of Min genes. The Min system is hierarchically assembled, with DivIVA recruiting MinJ, which then recruits MinD (62). In agreement with that, we saw a loss of polar and septal msfGFP-MinD localization (BHF025 and BHF026) when we knocked out *minJ* or *divIVA*, which we show in a Δ*minJ* background (BHF069) in Fig. S5, where *minC* was also knocked out to achieve comparable cell length distributions. Instead, loss of DivIVA or MinJ leads to a dispersed MinD localization with a weak enrichment of MinD around the cell center and a depletion at the cell poles in short cells (Fig. S5). Loss of polar and septal localization was also observed for MinJ-msfGFP upon knocking out *divIVA* (BHF032), further corroborating that DivIVA/MinJ complexes are required for controlled MinD localization. Therefore, we did not include these strains in the FRAP analysis. When *minCD* was knocked out in a strain expressing MinJ-msfGFP, the half-time recovery in FRAP dropped from 62 s to 30 s (Fig. 2; Table 3; Fig. S6). This behavior is in line with a direct interaction between the two proteins. We cannot exclude that the phenotype itself impacts the dynamic behavior of MinJ, since cells are elongated and often redivide after successful cytokinesis (65). When *minCD* was knocked out in a DivIVA-GFP-expressing strain (BHF040), however, we could not see any significant difference in fluorescence recovery. Since there is no direct interaction, DivIVA dynamics do not seem to be affected by MinCD directly or indirectly, which includes the effects of the phenotype of elongated cells. In contrast to that, knocking out *minJ* sped up recovery of DivIVA-GFP (BHF041) significantly, with a recovery time less than half of the wild type, which was also true for DivIVA-mNeonGreen (BHF027) (Table 3; Fig. S6). This result is consistent with a direct interaction. Interestingly, there was also an impact on the mobile fraction, which decreased from around two-thirds to roughly 40% to 50% in both strains. Thus, dynamics are modulated by complex formation reflecting the expected protein hierarchy. MinD recruitment to midcell is fully dependent on DivIVA/MinJ. Since these proteins are relocating only to late stages of septum development, e.g., after a cross wall has started to form, we argue that this geometric change in the cell is important to redistribute MinD from the poles to midcell and establish a new dynamic steady state at the septum/new pole. This localization of MinD at midcell is lost if either DivIVA or MinJ is deleted, or MinD ATPase activity is abolished, as it can be observed in the G12V and K16A ATPase mutants of MinD (81). Thus, maintenance of a steady gradient requires ATPase activity and is therefore similar to the E. coli system. Therefore, we aimed to support this hypothesis by mathematical modeling to further understand the observed dynamics.

10.1128/mBio.00296-21.6 FIG S5 Microscopic analysis of msfGFP-MinD in a Δ*minCJ* background (BHF069) reveals MinJ-independent MinD accumulation around midcell. (a) Averaged fluorescence (a.u.) of msfGFP-MinD in short (<5 μm, blue) and long (>5 μm, red) cells along the longitudinal cell axis of BHF069 (Δ*minCJ*) cells (*n* = 329 cells). Cell length was chosen according to the frequent appearance of a division septum in cells longer than 5 μm. Fluorescence values of profiles were normalized in length and fluorescence intensity per cell. The resulting values were then binned (bin = 0.05 μm). (b) Demograph showing fluorescence profiles of cells expressing msfGFP-MinD in strain BHF069 (Δ*minCJ*) along the longitudinal cell axes, sorted by length. Fluorescence intensities are illustrated relative to maximal intensity values per cell by a color gradient ranging from dark red (low intensities) over orange (intermediate) to white (high intensities). The blue dashed box indicates separation between short and long cells, separated at a 5-μm cell length. Short and long cells are each represented by a cell on the right, respectively (green, msfGFP-MinD; red, Nile red). Scale bar, 2 μm. Download FIG S5, TIF file, 0.6 MB. Copyright © 2021 Feddersen et al. 2021 Feddersen et al. https://creativecommons.org/licenses/by/4.0/ This content is distributed under the terms of the Creative Commons Attribution 4.0 International license.

10.1128/mBio.00296-21.7 FIG S6 Representative microscopy images of FRAP analysis of Min proteins in different knockout backgrounds. (a) MinJ-msfGFP expressed in Δ*minCD* background (BHF015), and DivIVA-GFP expressed in Δ*minCD* (BHF040), Δ*minJ* (BHF041), and Δ*minCDJ* (BHF042) backgrounds. Images were taken before bleaching of the indicated spot with a 488-nm laser pulse, directly after bleaching, and after recovery of fluorescence. Scale bars, 2 μm. (b) Representation of the normalized fluorescence recovery in the green channel over time. *T*_1/2_ is time when fluorescence recovery reaches half height of total recovery, indicated on the graph with a dashed square. The red line represents measured values, the black line represents fitted values. Download FIG S6, TIF file, 2.4 MB. Copyright © 2021 Feddersen et al. 2021 Feddersen et al. https://creativecommons.org/licenses/by/4.0/ This content is distributed under the terms of the Creative Commons Attribution 4.0 International license.

### Theoretical model for MinD dynamics in B. subtilis.

Previous theoretical analyses of the Min system in B. subtilis using quantitative mathematical models are sparse. To our knowledge, there is actually only a single theoretical study that has investigated a mechanism for the polar localization of proteins (82). In this work, the coupled dynamics of DivIVA and MinD are modeled by a reaction-diffusion system in one spatial dimension. Both MinD and DivIVA are considered to diffuse on the membrane and in the cytosol and cycle between these two compartments by attachment and detachment processes. Membrane-bound MinD is assumed to be stabilized through DivIVA, and hence its role is quite different from that of MinE, which destabilizes membrane-bound MinD. Moreover, it was argued that DivIVA requires the presence of MinD for membrane binding (82), specifically, that DivIVA binds to and then stabilizes the edges of MinD clusters. Note that this assumption is no longer valid, as more recent studies have shown that DivIVA can directly bind the membrane. Since the model was studied in one spatial dimension, the author accounted for geometric effects only implicitly by reducing the MinD attachment rate near the cell poles. The importance of ATP binding and hydrolysis on MinD activity has been discussed but was disregarded in the model, as explicit coupling between cytosol and membrane (bulk-boundary coupling) was not considered. In summary, the model was a first and important theoretical analysis dissecting the relative roles of MinD and DivIVA as well as their interplay in shaping protein localization in B. subtilis.

Here, on the basis of previous theoretical studies of intracellular protein dynamics (32, 34, 36, 83), we propose a minimal reaction-diffusion system to model Min localization in B. subtilis. Building on the idea of geometry sensing put forward previously (83), our model provides a possible mechanism for how proteins sense cell geometry. This mathematical analysis shows that Min polarization and localization are established through a highly dynamic process driven by the ATPase activity of MinD. This implies that Min protein gradients are maintained by genuine nonequilibrium processes and not by thermodynamic binding (chemical equilibrium) of Min proteins to a DivIVA template at the cell poles (3, 8).

**FIG 3:**
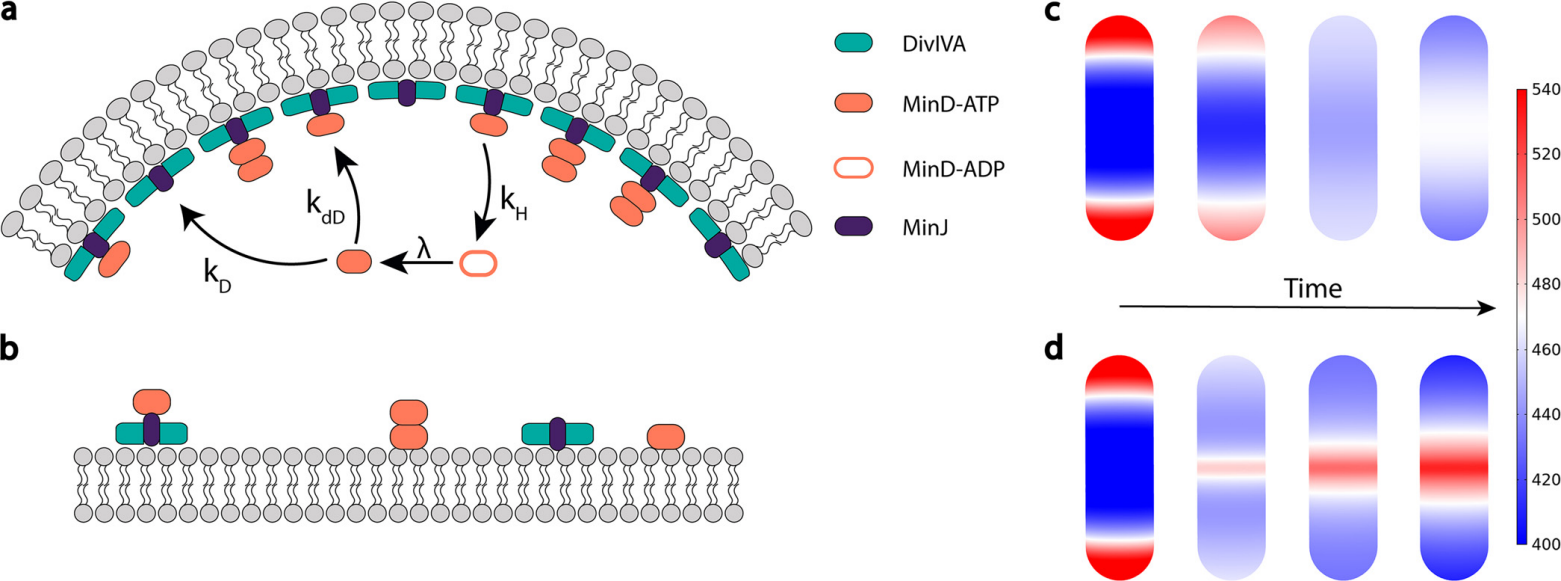
Model and simulation of the Min system in B. subtilis. (a) The geometry sensing protein DivIVA (green) preferentially localizes to regions of highest negative curvature and stabilizes MinJ (purple) to these regions. Membrane-bound DivIVA acts as a template for MinD recruitment of cytosolic MinD-ATP (orange) facilitated through MinJ. MinD-ATP binds to the membrane with a rate, $k_{D}$, and recruits cytosolic MinD-ATP with a (space-dependent) recruitment rate, $k_{\mathrm{dD}}$, to the membrane. Membrane-bound MinD is stabilized by MinJ-DivIVA complexes, which is reflected in a space-dependent detachment rate, $k_{\text{det}}$. After detachment, MinD is in a hydrolyzed state, MinD-ADP, and can rebind to the membrane only after nucleotide exchange with a rate λ. (b) MinD binds to flat membrane regions as well and recruits MinD-ATP from the cytosol. Binding to flat regions is, however, less favored, due to the lower concentration of MinJ-DivIVA complexes. (c) Simulation of the reaction-diffusion model in a 3D rod-shaped cell; shown is the membrane-bound MinD density distribution. As the initial condition, we take the steady-state distribution of the scenario where DivIVA is localized at the poles (left figure). At simulation start, we assume that MinD is losing its affinity to the poles by making the recruitment and detachment rate uniform on the entire cell membrane (this is, for example, the case at the onset of septum formation). From left to right, the time evolution of membrane-bound MinD is shown, where the far-right side shows the final steady-state density distribution. We find that polar localization of MinD becomes unstable and that the proteins preferentially localize at the cell center. (d) To test whether MinD can be localized at midplane through MinJ-DivIVA complexes after septum formation, we took the same initial condition as described for panel c and enhanced recruitment and decreased detachment near midcell. We find that MinD can sharply localize at the septum.

We study protein dynamics in realistic three-dimensional (3D) cellular geometry, where proteins cycle between cytosol and membrane, and MinD diffuses with diffusion constants $D_{D}=16{\text{ μm}}^{\text{2}}\;/\text{ s}$ and $D_{d}=0.06{\text{ μm}}^{\text{2}}\;/\text{ s}$ in the cytosol and on the membrane, respectively. We consider fully resolved dynamics of MinD (including its ATPase cycle). The biochemical reaction scheme, illustrated in Fig. 3a and b, is based on the following molecular processes: (i) attachment to and detachment from the membrane with rates $k_{D}=0.068\text{ μm/s}$ and ${\tilde{k}}_{H}=0.1\;s^{-1}$ respectively; (ii) a nonlinear recruitment process of cytosolic MinD by membrane-bound MinD with rate ${\tilde{k}}_{\mathrm{dD}}=0.04{\text{ μm}}^{\text{2}}\;/\text{ s}$; (iii) after detachment from the membrane, MinD is in an ADP-bound state and can rebind to the membrane only after nucleotide exchange, which occurs at rate $\lambda =6{\text{ s}}^{-1}$. The protein numbers and membrane diffusion of MinD were extracted from our measurements (Tables 2 and 3; see Table S1 in the supplemental material), and the values for the kinetic parameters (rate constants) were estimated from previous work on protein pattern formation (32, 34, 36, 83).

10.1128/mBio.00296-21.1 TABLE S1 Kinetic rate constants for the MinD dynamics. The membrane diffusion coefficient, MinD protein density, cell length, and cell width were chosen in accordance with our experimental data. The bulk diffusion coefficient, attachment rate, hydrolysis rate, and nucleotide exchange rate were estimated from previous approaches for intracellular protein dynamics. Download Table S1, DOCX file, 0.02 MB. Copyright © 2021 Feddersen et al. 2021 Feddersen et al. https://creativecommons.org/licenses/by/4.0/ This content is distributed under the terms of the Creative Commons Attribution 4.0 International license.

Since the above reaction scheme contains only the attachment and detachment kinetics of MinD, one would intuitively expect that the steady-state MinD membrane density distribution is spatially uniform. Interestingly, from finite element simulations (see Materials and Methods), we find that the steady-state density distribution of membrane-bound MinD is not homogeneous but is nonuniform along the whole cell body and with a weak maximum at midcell (Fig. 3c, right figure), comparable to our observations *in vivo* (Fig. S5). The reason for this unexpected spatial localization of MinD is a purely geometric effect suggested previously (83). For a better understanding of our following arguments, let us briefly summarize the core results of this study. Due to the curvature at the poles, the effective “hitting frequency” (attachment rate) of active MinD-ATP becomes larger in these regions, which initially leads to an accumulation of MinD-ATP at the poles. However, upon detachment, MinD is in an inactive MinD-ADP state and first needs to exchange its nucleotide in order to rebind to the membrane. Hence, during this time, one can define a characteristic length scale of $l=\sqrt{D_{D}/\lambda }$ (see Materials and Methods), during which inactive proteins travel in the cytosol until they become able to rebind to the membrane. For our parameter choice, we have l≈1.6 μm, which corresponds roughly to half the typical size of a B. subtilis cell (Table S1; and see Materials and Methods). Therefore, due to the curved cell geometry, MinD-ATP is depleted at the poles, resulting in an accumulation of MinD-ATP near midcell. To test this prediction, a strain expressing msfGFP-MinD in a *minJ* background was created (BHF069). Furthermore, we knocked out *minC* in this strain to partially account for the shifted cell length distribution of a *minJ* background. As predicted through the model, we found a clear maximum of msfGFP-MinD at midcell, when cells did not yet start to form a septum (Fig. S5), indicated by their size (<5 μm). Longer cells (>5 μm) often start to divide at midcell, thereby creating a membrane curvature that affects distribution of msfGFP-MinD. In these cells, the concentration is highest in the center of both cell halfs (Fig. S5). This finding also highlights the importance of realistic 3D simulations, as geometric sensing would be absent in simplified 1D systems.

As already outlined in the previous sections, experimental studies have shown that DivIVA binds preferentially to regions of high negative membrane curvature and that MinJ localization is dependent on the presence of DivIVA (61, 62). MinD does not interact with DivIVA directly but through MinJ, which is known to act as an intermediary between DivIVA and MinD (62). Furthermore, our experiments suggest that DivIVA-MinJ complexes act as a spatial template for MinD binding. This suggests that the effective role of DivIVA and MinJ on MinD binding can be summarized in spatially varying values of the MinD recruitment and detachment rate, where the recruitment rate is larger in the presence of DivIVA-MinJ complexes (cell poles and septum) and smaller in the remaining part of the cell. Similarly, the detachment rate is lower in the presence of DivIVA-MinJ complexes (cell poles and septum) and higher otherwise. Intuitively, one would then expect that MinD localizes to those regions where the recruitment and detachment rate are altered, as this would effectively result in a higher binding rate of MinD.

To put this idea into test, we first incorporated space-dependent recruitment and detachment rates of MinD at membrane areas with a negative curvature; for details, see Materials and Methods (Fig. S7). Under the above conditions, MinD accumulates at both cell poles in a dynamic equilibrium state, with proteins constantly cycling between cytosol and membrane (Fig. 3c, left figure). In contrast, in the absence of preferential attachment at the cell poles facilitated by DivIVA-MinJ complexes (i.e., by employing uniform rates), polar localization of MinD becomes unstable and the proteins become preferentially localized in the cell center (again in a dynamic equilibrium state). The underlying reason is the geometric effect as explained above. To appreciate this result, note that this effect alone could explain the redistribution of MinD from the cell poles to midcell at the onset of cytokinesis (initiated by the redistribution of DivIVA to the septum, which would have a higher curvature than the cell poles).

10.1128/mBio.00296-21.8 FIG S7 Geometry for the simulation of the model. (a) Sketch of the simulation geometry (spherocylinder). (b) Polar localization is achieved by setting *α* = 4 and *β* = 3 at the poles (green area), and *α* = *β* = 1 for the remaining part of the geometry. (c) Localization at the septum is achieved by setting *α* = 4 and *β* = 3 in a narrow region at midcell (green) and otherwise *α* = *β* = 1. Download FIG S7, TIF file, 0.2 MB. Copyright © 2021 Feddersen et al. 2021 Feddersen et al. https://creativecommons.org/licenses/by/4.0/ This content is distributed under the terms of the Creative Commons Attribution 4.0 International license.

Next, we tested whether MinD can be localized at midplane in the presence of DivIVA-MinJ complexes once a septum has formed there. Indeed, emulating the presence of these complexes by an enhanced recruitment and detachment rate localized at the septum, our simulations show that MinD becomes sharply localized at midplane following the transfer of DivIVA-MinJ complexes from the poles to the septum (Fig. 3d). The width of the MinD distribution at midcell is determined by the interplay between membrane diffusion and localized recruitment of MinD at the septum (see Materials and Methods).

### Single-molecule resolution of the Min system reveals cluster formation.

**FIG 4:**
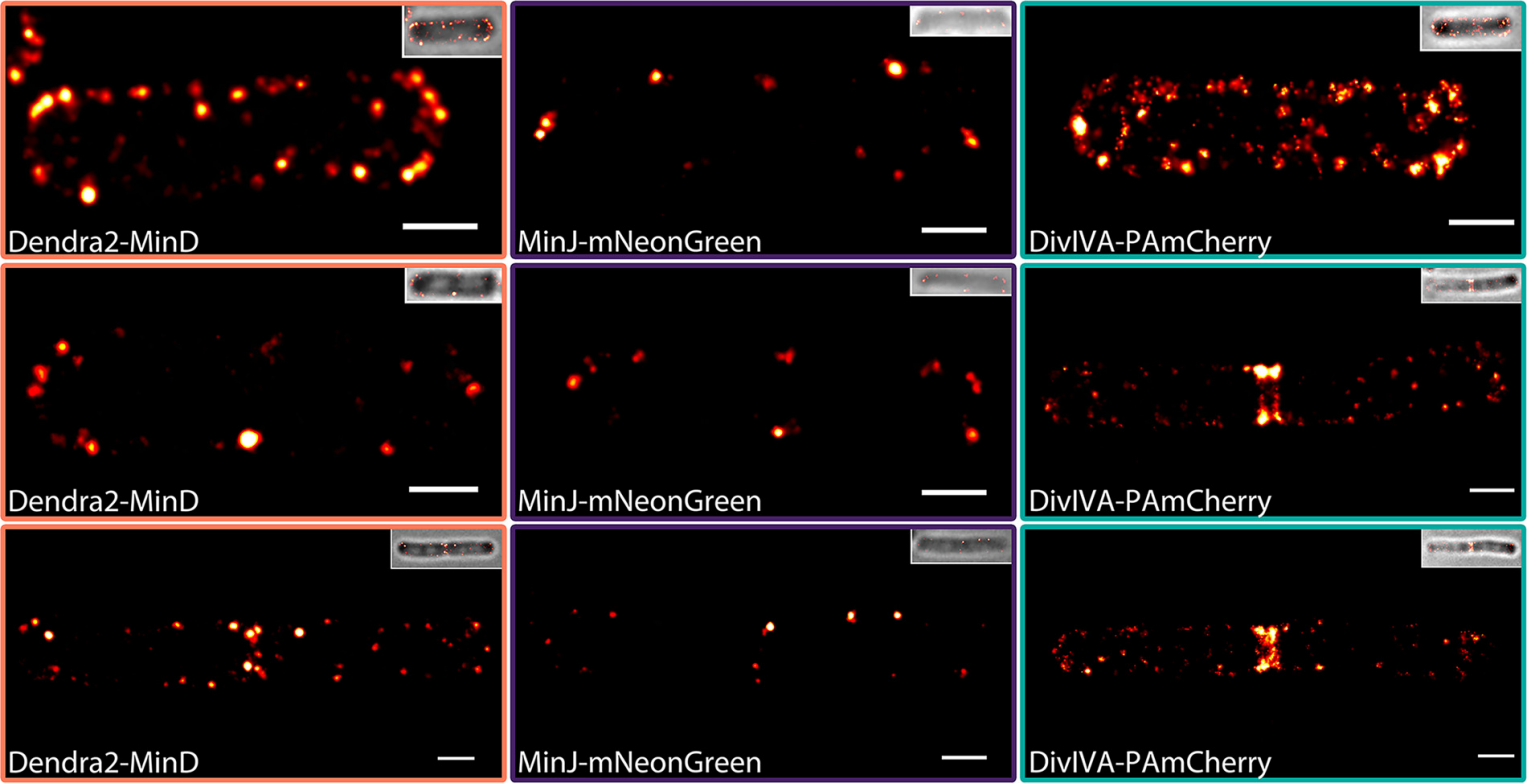
PALM imaging of strains expressing Dendra2-MinD, MinJ-mNeonGreen, and DivIVA-PAmCherry. Representative PALM images of Dendra2-MinD (BHF011), MinJ-mNeonGreen (JB40), and DivIVA-PAmCherry (JB37) expressing cells at different divisional states are shown. Upon formation of a division site, DivIVA, MinJ, and MinD partially relocalize from the poles to the division septum, where they reside after successful cytokinesis. Samples were fixed prior to imaging; every image represents a different cell. Scale bar, 500 nm.

Next, we wanted to test these theoretical predictions concerning a dynamic steady state of MinD proteins experimentally, using single-molecule resolution microscopy. In contrast to a stationary bipolar gradient of Min proteins from the cell poles, as described before (3, 8, 57, 58) based on a simple thermodynamic binding of Min proteins to a DivIVA/MinJ template, we expect a dynamic relocalization of Min proteins from the cell pole to the septum. This dynamic steady state would reveal Min components along the entire membrane, including the lateral sites at any time. To achieve the highest possible resolution, we used photoactivated light microscopy (PALM). Accordingly, strains expressing Dendra2-MinD (BHF011), MinJ-mNeonGreen (JB40), and DivIVA-PAmCherry (JB37) were utilized. While Dendra2 and PAmCherry are photoswitchable or photactivatable FPs that can be converted from green to red or activated with UV light, respectively, and are hence well suited for PALM (68), mNeonGreen can be used for PALM because of its innate capability to photoswitch (70). However, mNeonGreen presents some challenges in comparison to classical photoactivatable FPs, as it cannot be prebleached and therefore requires more postprocessing to reach satisfying artifact-free molecule localizations (75). Nevertheless, all three strains could be successfully imaged in fixed cells with average precisions of 25 to 30 nm (Fig. 4) using appropriate filter settings (see Table 7).

Importantly, we observed that all Min proteins not only localized to the cell poles but also as clusters along the membrane and with some apparent cytoplasmic localizations. These protein accumulations were mainly seen along the membrane for MinJ (Fig. 4, middle panels), while a fraction of MinD and DivIVA could be observed in the cytosol (Fig. 4, left and right panels). The high abundance of these protein accumulations indicates that recruitment of MinD and DivIVA by existing clusters progresses at higher rates than individual membrane binding, which is also reflected in the proposed mathematical model. Double rings of MinJ and DivIVA have been reported previously in 3D structured illumination microscopy (77), which could be observed in late divisional cells in PALM as well (Fig. 4, middle and bottom panels). The active enrichment at the young cell pole is consistent with the theoretical model described above and with a role of the Min system in regulation of cell division rather than protection of cell poles from aberrant cell division (65).

**FIG 5:**
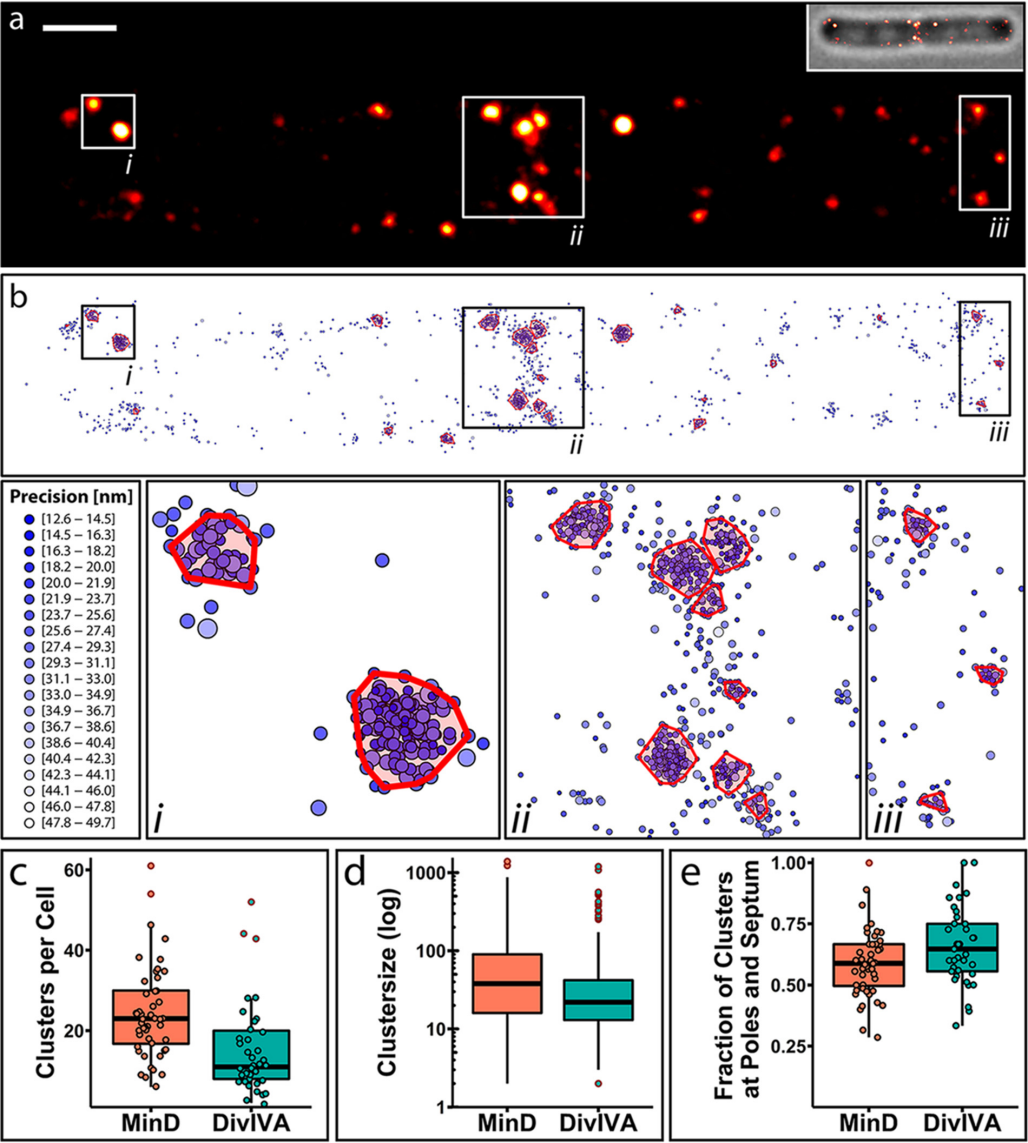
PALM imaging and representative cluster analysis of Dendra2-MinD and DivIVA-PAmCherry. (a) Representative PALM image of Dendra2-MinD (BHF011) in a cell in a late division state. Scale bar, 500 nm. (b) Cluster analysis of the same PALM data as shown in panel a with three highlighted regions (i, ii, and iii). Cluster analysis was performed in R using the OPTICS algorithm from the DBSCAN package. Every point indicates a single event and thus a Dendra2-MinD/DivIVA-PAmCherry protein, and precision is indicated by color and size of the circle. (c) Box plot of the number of clusters of Dendra2-MinD and DivIVA-PAmCherry per cell (MinD, *n*_cells_ = 48; DivIVA, *n*_cells_ = 37). (d) Box plot of the number of proteins per cluster; no jitter is shown due to the high sample number (Dendra2-MinD, *n*_clusters_ = 1,171; DivIVA-PAmCherry, *n*_clusters_ = 586). (e) Box plot of fraction of clusters localized at poles and septa per cell (MinD, *n*_cells_ = 48; DivIVA, *n*_cells_ = 37). Outliers in box plots are indicated by a red outline.

To get a deeper insight into the structure and distribution of the imaged proteins and to confirm clustering, a single-molecule point-based cluster analysis was performed for MinD and DivIVA (Fig. 5; Fig. S8). Unfortunately, MinJ-mNeonGreen imaging did not produce a sufficient number of events to be analyzed robustly (Fig. S8a), as MinJ expression levels are low in comparison and only a small fraction of mNeonGreen molecules blink reliably (75).

10.1128/mBio.00296-21.9 FIG S8 PALM imaging and representative cluster analysis of strain JB40 expressing MinJ-mNeonGreen and strain JB37 expressing DivIVA-PAmCherry. (a) PALM image of MinJ-mNeonGreen in a cell in a late division state. Scale bar, 500 nm. (b) Cluster analysis of the same PALM data with three highlighted regions (i, ii, and iii). Cluster analysis was performed in R using the OPTICS algorithm from the DBSCAN package. Every point indicates a single event and thus a MinJ-mNeonGreen protein; precision is indicated by color and size of the circle. (c) PALM imaging and representative cluster analysis of strain JB37 expressing DivIVA-PAmCherry. PALM image of DivIVA-PAmCherry in a cell in a late division state. Scale bar, 500 nm. (d) Cluster analysis of the same PALM data with three highlighted regions (i, ii, and iii). Cluster analysis was performed in R using the OPTICS algorithm from the DBSCAN package. Every point indicates a single event and thus a DivIVA-PAmCherry protein; precision is indicated by color and size of the circle. Download FIG S8, TIF file, 1.2 MB. Copyright © 2021 Feddersen et al. 2021 Feddersen et al. https://creativecommons.org/licenses/by/4.0/ This content is distributed under the terms of the Creative Commons Attribution 4.0 International license.

In total, we recorded 151,887 events in 48 cells for Dendra2-MinD, while 52,377 events of DivIVA-PAmCherry were recorded in 37 cells. When clusters with at least 10 molecules per cluster were identified, 55.61% (84,470) of all Dendra2-MinD events were organized in clusters, while 52.27% (27,379) of events of DivIVA-PAmCherry could be assigned to clusters. Thus, the average prevalence of clusters per cell was higher for MinD (24 clusters per cell) than for DivIVA (15 clusters per cell) (Fig. 5c). The size of these clusters varied greatly (Fig. 5d): an average number of 72 MinD proteins per cluster was determined, while the average number of DivIVA proteins per cluster was 47. However, we also observed some very large clusters with up to 1,390 MinD proteins and 1,198 DivIVA proteins, respectively. Analysis of the relative position of all clusters per cell revealed a high tendency for clusters to form around poles and septa (Fig. 5e), where around two-thirds of DivIVA clusters (66%) and more than half of MinD clusters (59%) were observed, while the rest was found along the lateral membrane or in the cytosol. This correlates well with the idea that most of MinD is recruited to negative membrane curvature (poles and septum) by DivIVA via MinJ. MinD also binds to flat membrane areas, where it recruits more MinD from the cytosol. This is less favored due to the lower concentration of MinJ-DivIVA complexes, which is reflected in our simulations and data. Our data also reveal that MinD and DivIVA seem to accumulate, and cytosolic proteins therefore have a higher tendency to bind to existing clusters than to free membrane surfaces. We did not observe a large proportion of MinD dimers and also no homogeneous binding of MinD or DivIVA to the membrane.

## DISCUSSION

The Min reaction network has been extensively studied in various organisms (8, 84). In E. coli, it was found to be a highly dynamic and self-organizing system capable of pole-to-pole oscillation, a prime example for intracellular protein pattern formation (36). The two core components in this network, MinE and MinD, cycle between membrane and cytosol and are sufficient to induce robust protein patterns both *in vivo* and *in vitro* (19, 20, 29, 85, 86). Therefore, it has been puzzling that the Min system in B. subtilis was described to form a rather stationary bipolar gradient from poles to midcell, although MinC and MinD are well conserved. The differences are mainly accredited to the absence of MinE, which stimulates ATP hydrolysis and thus membrane detachment of MinD. Instead, the curvature-sensing DivIVA was found to recruit MinCDJ to the negatively curved poles. However, MinC has been shown to dynamically relocalize to midcell prior to division in fluorescence microscopy (67), and the same study highlights open questions in the current Min model for B. subtilis, pointing out that earlier studies were conducted using strains that artificially overexpress Min network components, thereby possibly masking dynamic populations.

In this study, we analyzed protein dynamics of the B. subtilis Min system based on experiments conducted with native expression levels of fluorescently labeled Min components. First, we found all components to be highly dynamic. MinD had the shortest recovery time of the three investigated proteins, while MinJ and DivIVA both had considerably slower recovery times than MinD but in a similar range when compared to each other (Table 3). Similar tendencies were detected when mobile and immobile fractions were compared, where MinD had the highest mobile fraction, with almost 80% of the protein taking part in the recovery. With diffusion coefficients between 0.057 μm^2^/s and 0.0034 μm^2^/s, the three proteins were found in an expected range for membrane (-associating) proteins in bacteria (87). Considering the nature of DivIVA, which binds to the membrane and stabilizes itself at negative curvature, and MinJ as an integral membrane protein, it is not surprising that the cytosolic MinD is around 10-fold faster in recovery. This observation leads to the speculation of a relatively fast exchange of membrane-bound MinD proteins at the division septum, considering relatively high total protein quantities (Table 2) in combination with a large mobile fraction and fast recovery when bleaching these sites. DivIVA total protein numbers were found to be around half of MinD, while MinJ was by far the least abundant Min component. These findings correlate with the corresponding fluorescence intensity and appearance when imaging the respective Min proteins tagged with the same fluorophore during mid-exponential growth (for examples, see Fig. S1 in the supplemental material).

Moreover, knocking out single or multiple components had an impact on the dynamics of the respective upstream recruiting factor, validating interactions between MinD and MinJ and between MinJ and DivIVA, respectively, that were observed in genetic studies previously (62, 63). Based on this interaction network and the respective protein behaviors in combination with the knowledge gained from the E. coli Min system, a mathematical model was designed.

We propose a minimal reaction-diffusion model that correctly reproduces qualitative features of MinD localization in B. subtilis. We extracted the parameters for the model from our measurements (protein numbers and diffusion coefficients) (Tables 2 and 3) and from previous work on intracellular protein pattern formation (32, 34, 36, 83). The basic assumption of the model is that DivIVA acts as a spatial template for MinJ and MinD, which we accounted for implicitly through a space-dependent recruitment and detachment rate for MinD. From the computational analysis of the model (finite element method [FEM] simulations), we found that localization of MinD to the poles or the division site corresponds to a dynamic equilibrium state of the reaction-diffusion equation. Further, our results show that a geometric effect alone is sufficient to guide MinD to the division site, therefore highlighting the importance of realistic 3D models.

Our model can be straightforwardly extended to include the explicit dynamics of DivIVA and MinJ. As the exact reaction network of the Min system in B. subtilis remains elusive, a theoretical model could help in identifying the essential components of Min dynamics. By following the same approach as for the E. coli Min system, reconstitution of the Min system *in vitro* would also help to dissect the complexity of the system and to make the comparison between experiments and theory even more feasible. We believe that our theoretical approach may serve as a basis for future studies addressing protein dynamics in B. subtilis.

We note that the observed dynamics are not compatible with a division site selection system, because ongoing division is needed for correct localization and dynamics of the Min system in B. subtilis. This is in line with data obtained by Elizabeth Harrýs lab showing that deletion of Min proteins does not abolish midcell positioning of the Z-ring in B. subtilis (88) and our own data describing reduced disassembly of Z-rings in the absence of the Min system (65). The model we propose includes a yet unknown protein or mechanism that stimulates MinD-ATP hydrolysis. The uniform hydrolysis rate $k_{H}$ in our model was predicted to be similar to that of the closely related MinD in E. coli, which is stimulated by MinE (25, 29). The responsible protein or mechanism has yet to be elucidated, but the presence of cytosolic and membrane-bound MinD fractions and their respective dynamics as well as the well-conserved ATPase domain argue very convincingly for its existence.

Additionally, we investigated the Min components with single-molecule resolution, revealing a strong tendency for cluster formation, and these clusters are also found at the lateral sides of the cell membrane. The lateral Min assemblies have not been resolved by conventional light microscopy images, and hence the idea of an exclusive polar Min assembly was manifested. Clusters of MinD and DivIVA are indeed frequently observed close to poles and midcell. In accordance with the mathematical model, we hence hypothesize that a fraction of MinD will diffuse away from these primary binding sites after recruitment. Most of this fraction will quickly unbind the membrane due to the lack of stabilization and will be recruited again by DivIVA-MinJ to either pole or septum clusters. Proteins that are part of a cluster will show less exchange or dynamic behavior, further decreasing toward the center, as is typically observed in protein clusters (89). This mechanism could tightly regulate spatiotemporal localization of MinCD and, likewise, aid in transitioning from polar localization to septal localization rapidly upon septum formation, as DivIVA and MinJ would transition to the septum first. Since the current view on the task of the Min system in B. subtilis proposes a role downstream of cell division, all components need to be concentrated at the septum in time to inhibit a second round of division by promoting the disassembly of the division apparatus (65).

This study provides a model of the Min protein dynamics in B. subtilis that makes testable predictions. It emerges that the Min systems in B. subtilis and E. coli are not so fundamentally different as initially thought. Future research will therefore address the unsolved question of how MinD ATPase activity is triggered in B. subtilis. Furthermore, the influence of membrane binding of DivIVA and MinD requires a closer look to gain quantitative data for a refined mathematical model.

## MATERIALS AND METHODS

### Bacterial strains, plasmids, and oligonucleotides.

The oligonucleotides, plasmids, and strains used in this study are listed in Tables 4 to 6, respectively. E. coli NEB Turbo was used to amplify and maintain plasmids.

**TABLE 4:** Oligonucleotides used in this study

Oligonucleotide name	Sequence (5′ to 3′)
bsarem1	TTTGGTCTCAGGTTCTCGCGGTATCATTGCAGC
bsarem2	TTTGGTCTCAAACCACGCTCACCGGCTCCAG
HF0061	GTCGGTCTCAACTAGAATTCGTAATCATGGTCATAGCTG
HF0062	CTCGGTCTCATCGGAAGCTTGGCACTGGC
HF0037	TATGGTCTCCCCGAGTTCATTCTATTGACAGTGAAGTC
HF0038	CTAGGTCTCTCTCCTTCACATTCCTCCCTCAAG
HF0040	AATGGTCTCTGGAGGGGTGAAAGGATGTACTTA
HF0041	TTTGGTCTCGCGAATAATTGAGAGAAGTTTCTATAG
HF0042	GGAGGTCTCTTTCGATGAACACCCCGGGAATTAAC
HF0043	CACGGTCTCCCATTCCACACCTGGCTGGGCAGG
HF0044	ACGGGTCTCAAATGGGTTGGGTGAGGCTATCGTAATAAC
HF0045	CGGGGTCTCTTAGTCAATATTTTCCTCTTGCTCCAGC
HF0065	GGAGGTCTCTTTCGATGGGTACCCTGCAGATG
HF0066	CACGGTCTCCCATTTTTGTAGAGCTCATCCATGC
G40	CTAGGTCTCTCCGATGTCGGATTTGGACA
G41	TATGGTCTCCCTCCTGATCCCGAAGCGAC
HF0029	AATGGTCTCTGGAGGGATGGGTACCCTGCAGATG
HF0030	TTTGGTCTCGCGAATTTGTAGAGCTCATCCATGC
G20	AATGGTCTCTGGAGGGATGAACACCCCGGGAATTAAC
G21	TTTGGTCTCGCGAATTACCACACCTGGCT
G36	GGAGGTCTCTTTCGGGGTGAAAGGATGTACTTA
G37	CACGGTCTCCCATTTAATTGAGAGAAGTT
G42	ACGGGTCTCAAATGGGAAGGCAGCCCGGCACCGCAGG
G43	CGGGGTCTCTTAGTCCATGATGGCTGGTG
HF0077	AATGGTCTCTGGAGGGATGGTGAGCAAGGGCG
HF0078	TTTGGTCTCGCGAATTACTTGTACAGCTCGTCCATG
G32	ACGGGTCTCAAATGGGATTCTCTGATTATCT
G33	CGGGGTCTCTTAGTATCGGGAAATCTGTT
G34	CTAGGTCTCTCCGAGAATTCCTAGCCCAAGTCAG
G35	TATGGTCTCCCTCCTTCCTTTTCCTCAAA
HF0206	TATGGTCTCCCCGAGTTAACCGTGACGTGC
HF0207	CTAGGTCTCTCTCCAATATTCACCTCAACAACATAC
HF0203	AATGGTCTCTGGAGTACCGTTCGTATAGCATAC
HF0204	TTTGGTCTCGCGAATCTACCGTTCGTATAATG

**TABLE 5:** Plasmids used in this study

Plasmid	Characteristics	Reference or source
pUC18	*lacZα*, pMB1 *ori*, *bla* (Ap^r^)	99
pUC18mut	pUC18 with mutated BsaI site in *bla*	Laboratory collection
pDendra2-N	pUC *ori*, SV40 *ori*, PCMVIE, *aph3-A3*	Evrogen
pNCS-mNeonGreen	pUC *ori*, SV40 *ori, bla* (Ap^r^)	Allele Biotechnology
pUC57-DivIVAd34-mNG	pUC57-BsaI-free*, bla* (Ap^r^), *divIVAΔ34-mNeonGreen*	Synthesized by Biocat
pHJS105	*amyE* integration vector containing P*xyl-msfGFP-MCS, spc bla*	100
pHF01	pUC18mut*-minDup-aad9-Dendra2-minD*	This study
pHF02	pUC18mut*-minDup-aad9-msfGFP-minD*	This study
pHF03	pUC18mut*-minJ-msfGFP-aad9-minJdown*	This study
pHF04	pUC18mut*-minJ-mNG-aad9-minJdown*	This study
pHF05	pUC18mut*-divIVA-mNG-aad9-divIVAdown*	This study
pHF06	pUC18mut*-minJ-Dendra2-aad9-minJdown*	This study
pHF07	pUC18mut*-divIVA-Dendra2-aad9-divIVAdown*	This study
pHF08	pUC18mut-*divIVAΔ34-mNG-aad9-divIVAdown*	This study
pHF09	pUC18mut-*minCup-aph3-A3—aad9*	This study

**TABLE 6:** Strains used in this study

Strain	Relevant features or genotype	Reference or source
B. subtilis		
168	*trpC2*	Laboratory collection
3309	*minCD*::*aph3-A3*	Wu and Errington, 2004 (12)
RD021	*minJ*::*tet*	Bramkamp et al., 2008 (62)
4041	*divIVA*::*tet*	Bramkamp et al., 2008 (62)
SB075	*minCD*::*erm minJ*::*tet*	Laboratory collection
BHF011	*minD*::*aad9-Dendra2-minD*	This study, pHF01→168
BHF017	*minD*::*aad9-msfGFP-minD*	This study, pHF02→168
BHF025	*minD*::*aad9-msfGFP-minD minJ*::*tet*	This study, pHF02→RD021
BHF026	*minD*::*aad9-msfGFP-minD divIVA*::*tet*	This study, pHF02→4041
JB038	*minJ*::*minJ-Dendra2-aad9*	This study, pHF06→168
BHF007	*minJ*::*minJ-msfGFP-aad9*	This study, pHF03→168
BHF015	*minJ*::*minJ-msfGFP-aad9 minCD*::*aph3-A3*	This study, pHF03→3309
BHF032	*minJ*::*minJ-msfGFP-aad9 divIVA*::*tet*	This study, pHF03→4041
JB40	*minJ*::*minJ-mNeonGreen-aad9*	This study, pHF04→168
JB36	*divIVA*::*divIVA-Dendra2-aad9*	This study, pHF07→168
BHF028	*divIVA*::*divIVA-mNeonGreen-aad9*	This study, pHF05→168
BHF036	*divIVA*::*divIVA-mNeonGreen-aad9 minCD*::*aph3-A3*	This study, pHF05→3309
BHF027	*divIVA*::*divIVA-mNeonGreen-aad9 minJ*::*tet*	This study, pHF05→RD021
BHF037	*divIVA*::*divIVA-mNeonGreen-aad9 minCD*::*erm minJ*::*tet*	This study, pHF05→SB075
1803	*divIVA*::*divIVA-GFP-cat*	Thomaides et al., 2001 (76)
BHF040	*divIVA*::*divIVA-GFP-cat minCD*::*aph3-A3*	This study, 1803→3309
BHF041	*divIVA*::*divIVA-GFP-cat minJ*::*tet*	This study, 1803→RD021
BHF042	*divIVA*::*divIVA-GFP-cat minCD*::*erm minJ*::*tet*	This study, 1803→SB075
BHF067	*divIVA*::*divIVAΔ34-mNG-aad9*	This study, pHF08→168
BHF069	*minD*::*aad9-msfGFP-minD minC*::*aph3-A3 minJ*::*tet*	This study, pHF09→BHF025
JB37	*divIVA*::*divIVA-PAmCherry-aad9*	Stockmar et al., 2018 (75)
E. coli		
NEB Turbo	F′ *proA*^+^*B*^+^ *lacI*^q^ Δl*acZM15*/*fhuA2* Δ(*lac-proAB*) *glnV galK16 galE15* R(*zgb-210*::Tn*10*)Tet^s^ *endA1 thi-1* Δ(*hsdS-mcrB*)*5*	New England Biolabs

### Strain construction: Golden Gate assembly.

Fragments for Golden Gate assembly were amplified from B. subtilis 168 (*trpC2*) genomic DNA or template plasmids via PCR with the respective primers containing directional overhangs (Table 4). The vector pUC18mut was also amplified via PCR to introduce BsaI restriction sites and allow subsequent digestion of circular PCR template with DpnI, which cuts only methylated DNA. Plasmid construction was verified via individual control digestion and DNA sequencing. Correct plasmids were transformed into B. subtilis 168 with the respective genetic background (Table 6) and selected for the introduced resistance (Table 5). Resistant candidates were confirmed by PCR and microscopy.

pHF01 (pUC18mut*-minDup-aad9-Dendra2-minD*) was created by a Golden Gate assembly of 5 fragments: (i) PCR with primers HF0061 and HF0062 with pUC18mut as the template (yielding a linear pUC18mut); (ii) PCR with primers HF0037 and HF0038 and 168 genomic DNA (containing the region upstream of *minD*); (iii) PCR with primers HF0040 and HF0041 and JB40 genomic DNA (containing the spectinomycin adenyltransferase gene *aad9*); (iv) PCR with primers HF0042 and HF0043 and pDendra2-N plasmid DNA (containing the *Dendra2* gene); (v) PCR with primers HF0044 and HF0045 and 168 genomic DNA (containing the N-terminal region of *minD*).

pHF02 (pUC18mut*-minDup-aad9-msfGFP-minD*) was created by a Golden Gate assembly of 5 fragments: (i) PCR with primers HF0061 and HF0062 with pUC18mut as the template (yielding a linear pUC18mut); (ii) PCR with primers HF0037 and HF0038 and 168 genomic DNA (containing the region upstream of *minD*); (iii) PCR with primers HF0040 and HF0041 and JB40 genomic DNA (containing the spectinomycin adenyltransferase gene *aad9*); (iv) PCR with primers HF0065 and HF0066 and pHJS105 (containing the *msfGFP* gene); (v) PCR with primers HF0044 and HF0045 and 168 genomic DNA (containing the N-terminal region of *minD*).

pHF03 (pUC18mut*-minJ-msfGFP-aad9-minJdown*) was created by a Golden Gate assembly of 5 fragments: (i) PCR with primers HF0061 and HF0062 with pUC18mut as the template (yielding a linear pUC18mut); (ii) PCR with primers G40 and G41 and 168 genomic DNA (containing the C-terminal region of *minJ*); (iii) PCR with primers HF0029 and HF0030 and pHJS105 (containing the *msfGFP* gene); (iv) PCR with primers G36 and G37 and JB40 genomic DNA (containing the spectinomycin adenyltransferase gene *aad9*); (v) PCR with primers G42 and G43 and 168 genomic DNA (containing the region downstream of *minJ*).

pHF04 (pUC18mut*-minJ-mNG-aad9-minJdown*) was created by a Golden Gate assembly of 5 fragments: (i) PCR with primers HF0061 and HF0062 with pUC18mut as the template (yielding a linear pUC18mut); (ii) PCR with primers G40 and G41 and 168 genomic DNA (containing the C-terminal region of *minJ*); (iii) PCR with primers HF0077 and HF0078 and pNCS-mNeonGreen DNA (containing the *mNeonGreen* gene); (iv) PCR with primers G36 and G37 and JB40 genomic DNA (containing the spectinomycin adenyltransferase gene *aad9*); (v) PCR with primers G42 and G43 and 168 genomic DNA (containing the region downstream of *minJ*).

pHF05 (pUC18mut*-divIVA-mNG-aad9-divIVAdown*) was created by a Golden Gate assembly of 5 fragments: (i) PCR with primers HF0061 and HF0062 with pUC18mut as the template (yielding a linear pUC18mut); (ii) PCR with primers G34 and G35 and 168 genomic DNA (containing the C-terminal region of *divIVA*); (iii) PCR with primers HF0077 and HF0078 and pNCS-mNeonGreen DNA (containing the *mNeonGreen* gene); (iv) PCR with primers G36 and G37 and JB40 genomic DNA (containing the spectinomycin adenyltransferase gene *aad9*); (v) PCR with primers G32 and G33 and 168 genomic DNA (containing the region downstream of *divIVA*).

pHF06 (pUC18mut*-minJ-Dendra2-aad9-minJdown*) was created by a Golden Gate assembly of 5 fragments: (i) PCR with primers HF0061 and HF0062 with pUC18mut as the template (yielding a linear pUC18mut); (ii) PCR with primers G40 and G41 and 168 genomic DNA (containing the C-terminal region of *minJ*); (iii) PCR with primers G20 and G21 and pDendra2-N plasmid DNA (containing the *Dendra2* gene); (iv) PCR with primers G36 and G37 and JB40 genomic DNA (containing the spectinomycin adenyltransferase gene *aad9*); (v) PCR with primers G42 and G43 and 168 genomic DNA (containing the region downstream of *minJ*).

pHF07 (pUC18mut*-divIVA-Dendra2-aad9-divIVAdown*) was created by a Golden Gate assembly of 5 fragments: (i) PCR with primers HF0061 and HF0062 with pUC18mut as the template (yielding a linear pUC18mut); (ii) PCR with primers G34 and G35 and 168 genomic DNA (containing the C-terminal region of *divIVA*); (iii) PCR with primers G20 and G21 and pDendra2-N plasmid DNA (containing the *Dendra2* gene); (iv) PCR with primers G36 and G37 and JB40 genomic DNA (containing the spectinomycin adenyltransferase gene *aad9*); (v) PCR with primers G32 and G33 and 168 genomic DNA (containing the region downstream of *divIVA*).

pHF08 (pUC18mut*-divIVAΔ34-mNG-aad9-divIVAdown*) was created by a Golden Gate assembly of 4 fragments: (i) PCR with primers HF0061 and HF0062 with pUC18mut as the template (yielding a linear pUC18mut); (ii) PCR with primers G34 and HF0078 and pUC57-DivIVAd34-mNG plasmid DNA (containing *divIVAΔ34-mNeonGreen*); (iii) PCR with primers G36 and G37 and JB40 genomic DNA (containing the spectinomycin adenyltransferase gene *aad9*); (iv) PCR with primers G32 and G33 and 168 genomic DNA (containing the region downstream of *divIVA*).

pHF09 (pUC18mut-*minCup-aph3-A3-aad9*) was created by a Golden Gate assembly of 4 fragments: (i) PCR with primers HF0061 and HF0062 with pUC18mut as the template (yielding a linear pUC18mut; (ii) PCR with primers HF0206 and HF0207 and 168 genomic DNA (containing the region upstream of *minC*); (iii) PCR with primers HF0203 and HF0204 and 3309 genomic DNA (containing the aminoglycoside-3′-phosphotransferase gene *aph3-A3*, conferring resistance to kanamycin); (iv) PCR with primers G36 and G37 and JB40 genomic DNA (containing the spectinomycin adenyltransferase gene *aad9*).

### Media and growth conditions.

B. subtilis was grown on nutrient agar plates using commercial nutrient broth and 1.5% (wt/vol) agar at 37°C overnight. To reduce inhibitory effects, antibiotics were used only for transformations and when indicated, since allelic replacement is stable after integration (chloramphenicol, 5 μg ml^−1^; tetracycline, 10 μg ml^−1^; kanamycin, 5 μg ml^−1^; spectinomycin, 100 μg ml^−1^; erythromycin, 1 μg ml^−1^).

For growth curves, B. subtilis was inoculated to an optical density at 600 nm (OD_600_) of 0.05 from a fresh overnight culture and grown in LB (lysogeny broth) (10 g liter^−1^ tryptone, 10 g liter^−1^ NaCl, and 5 g liter^−1^ yeast extract) at 37°C with aeration in baffled shaking flasks (200 rpm) to an OD_600_ of 1. Subsequently, cultures were diluted to an OD_600_ of 0.1 in fresh LB and measured every hour for at least 6 h.

For microscopy, B. subtilis was inoculated to an OD_600_ of 0.05 from a fresh overnight culture and grown in MD medium, a modified version of Spizizen minimal medium (90), at 37°C with aeration in baffled shaking flasks (200 rpm) to an OD_600_ of 1. MD medium contains 10.7 mg ml^−1^ K_2_HPO_4_, 6 mg ml^−1^ KH_2_PO_4_, 1 mg ml^−1^ Na_3_ citrate, 20 mg ml^−1^ glucose, 20 mg ml^−1^ l-tryptophan, 20 mg ml^−1^ ferric ammonium citrate, 25 mg ml^−1^ l-aspartate, and 0.36 mg ml^−1^ MgSO_4_ and was always supplemented with 1 mg ml^−1^ Casamino Acids. Subsequently, cultures were diluted to an OD_600_ of 0.1 in fresh MD medium and grown to an OD_600_ of 0.5 (exponential phase).

For epifluorescence and time-lapse imaging (e.g., FRAP), B. subtilis cells were mounted on prewarmed 1.5% MD agarose pads, sealed with paraffin, and incubated for 10 min at 37°C before microscopic analysis. When used, FM4-64 dye or Nile red was added to the agarose pad before polymerization (1 μM final concentration).

For PALM imaging, a 0.5-ml portion of B. subtilis cells was fixed by addition of formaldehyde (1.5% [wt/vol] final concentration) and incubated for 20 min at 37°C. Subsequently, cells were washed (1 min, 2,300 relative centrifugal force [rcf]), resuspended in fresh MD medium supplemented with 10 mM glycine to stop the cross-linking reaction, and incubated for 10 min at 37°C. Cells were then washed 2 more times with MD medium containing 10 mM glycine. In a final washing step, the pellet was resuspended in 50 μl of MD medium with 10 mM glycine to reach a higher cell density. Cells were mounted on chambered coverslips (μ-slide 8 well; Ibidi) containing 200 μl MD medium with 10 mM glycine, which were pretreated for 30 to 60 min with 0.1% poly-l-lysine and successively washed 3 times with MD medium containing 10 mM glycine. Furthermore, TetraSpeck microspheres (100 nm; ThermoFisher) were added at a dilution that results in about 3 to 10 beads per field of view. To help sedimentation of cells and beads and to reach a uniform attachment to the glass surface, the chambered coverslip was centrifuged at 3,400 rcf for 10 min in a bucket-swing rotor (Eppendorf).

### Typhoon imaging and Western blot analysis.

To confirm the presence of full-length protein fusions and for quantitative analysis, B. subtilis strains were inoculated from an overnight culture to an OD_600_ of 0.05 in the morning and grown to an OD_600_ of 0.5 in 10 ml LB medium (MD medium for quantitative studies) at 37°C. Cells were then diluted 1/10 and grown again to mid-exponential phase (OD_600_, 0.5). Cultures were centrifuged at 15,700 rcf for 1 min, washed once with lysis buffer (10 mM Tris, pH 7.5, 150 mM NaCl, 500 μM EDTA, 1 mM phenylmethylsulfonyl fluoride [PMSF]), and resuspended in lysis buffer with additional 10 mg/ml lysozyme (Sigma-Aldrich), 10 μg/ml DNase I (Roche), and 100 μg/ml RNase A (Roche), concentrating the sample to an OD_600_ of 30. After incubation at 37°C for 20 min, the sample was briefly vortexed to crack the remaining intact cells. Thirty microliters of sample was then mixed with 10 μl of 4× SDS-PAGE loading buffer (200 mM Tris-HCl [pH 6.8], 400 mM dithiothreitol [DTT], 8% SDS, 0.4% bromophenol blue, and 40% glycerol). For Typhoon imaging and subsequent Western blotting, either samples were incubated for 20 min at room temperature or, for some samples meant exclusively for Western blotting, they were incubated at 95°C for 10 min for full denaturation (indicated in Fig. S3 in the supplemental material). Ten or 20 μl of sample was then separated by SDS-PAGE in 12% Bis-Tris gels. For visualization of green fluorescent fusions, gels were imaged in a Typhoon Trio (GE Healthcare; photomultiplier voltage [PMT], 600 to 800; excitation, 488 nm; emission, 526 short pass filter [SP]). For Western blotting, proteins were blotted onto 0.2-μm-pore-size polyvinylidene difluoride (PVDF) membranes. Proteins were visualized via anti-mCherry (polyclonal), anti-mNG (monoclonal), or anti-Dendra (polyclonal) antibodies, respectively.

To quantify Dendra2 fusions of MinD, MinJ, and DivIVA via in-gel fluorescence, three biological triplicates were prepared and imaged as described above, while avoiding oversaturation. The total number of MinD molecules was taken from a publication that utilized targeted mass spectrometry to determine absolute protein amounts of B. subtilis at mid-exponential phase in minimal medium with glucose (78). Relative quantification was then performed using ImageJ by measuring and comparing intensities of the bands.

### Fluorescence microscopy.

For strain characterization, microscopy images were taken with a Zeiss Axio Observer Z1 microscope equipped with a Hamamatsu OrcaR^2^ camera using a Plan-Apochromat 100×/1.4 oil Ph3 objective (Zeiss). Dendra2, GFP, msfGFP, and mNeonGreen fluorescence was visualized with a 38 HE eGFP shift-free filter set (Zeiss), and FM4-64 membrane dye was visualized with a 63 HE mCherry filter set (Zeiss). The microscope was equipped with an environmental chamber set to 37°C. Digital images were acquired with Zen software (Zeiss).

For FRAP experiments, a Delta Vision Elite imaging system (GE Healthcare, Applied Precision) equipped with an InsightSSI illumination unit, an X4 laser module, and a CoolSnap HQ2 charge-coupled device (CCD) camera was used. Images were taken with a 100× oil PSF U-Plan S-Apo 1.4 numerical aperture objective. A four-color standard set InsightSSI unit was used with the following: excitation wavelengths for DAPI (4′,6-diamidino-2-phenylindole), 390/18 nm; FITC (fluorescein isothiocyanate), 475/28 nm; TRITC (tetramethyl rhodamine isocyanate), 542/27 nm; and Cy5, 632/22 nm; single band pass emission wavelengths for DAPI, 435/48 nm; FITC, 525/48 nm; TRITC, 597/45 nm; and Cy5, 679/34 nm; and a suitable polychroic for DAPI/FITC/TRITC/Cy5. GFP, msfGFP, and mNeonGreen were visualized using FITC settings and exposure times between 0.1 s (msfGFP, GFP) and 0.2 s (mNeonGreen). Bleaching was performed using a 488-nm laser (50 mW) with 10% power and a 0.005- to 0.01-s pulse. Frequency of acquisition and total amount of images were chosen according to the individual recovery times after initial testing with various settings.

Analysis of the images was performed using ImageJ 1.51 s. The corrected total cell fluorescence (CTCF) was calculated according to following formula: CTCF = integrated density − (area of selected cell × mean fluorescence of unspecific background readings) (91). For FRAP experiments, unspecific background was subtracted for every region of interest (ROI) (see above). The CTCF of the septa was divided by the CTCF of the whole cell to account for photobleaching during acquisition. The respective quotient of the unbleached spot was always set as 1 for normalization. Since B. subtilis keeps growing during time-lapse experiments like FRAP, the bleached spot moves in the field of view as cells elongate. Therefore, a macro in Fiji was created to dynamically follow and center the bleached spot through the frames of acquisition without any bias, which resulted in more precise FRAP curves. To determine half-time recovery and mobile/immobile fractions, the FRAP curve from the normalized recovery values was fitted to an exponential equation:

(1) $$I\left(t\right)=A(1\;-\;e^{-\tau t})$$

where I(t) is the normalized FRAP curve, *A* is the final value of the recovery, τ is the fitted parameter, and t is the time after the bleaching event. After determination of the fitted coefficients, they can be used to determine mobile (*A*) and immobile (1 − *A*) fractions, while the following equation was used to determine halftime recovery (equation 2):

(2) $$T_{\text{1/2}}=\frac{\ln\;0.5}{-\tau }$$

where $T_{\text{1/2}}$ is the halftime recovery and τ is the fitted parameter. Diffusion coefficients were then calculated with the following formula:

(3) $$D=(w^{2}/4T_{1/2})\times 0.88$$

according to Axelrod et al. (92), where D is the diffusion coefficient, w is the radius of the circular laser beam, and $T_{1/2}$ is the time when fluorescence recovery reaches half height of total recovery. To estimate the bleaching spot radius, cells expressing cytosolic GFP were fixed with 1.5% (vol/vol) formaldehyde as described above, mounted on agarose pads, bleached at laser powers of 10% to 100% in increments of 10%, and imaged right after bleaching. The radius was measured in ImageJ and averaged per triplicate to calculate the function of bleach radius over laser power. Graphs and statistics were created in R 3.3.1 (93) utilizing the packages ggplot2 (94) and nlstools (95). For measuring cell profiles, Fiji (ImageJ) was used, and a segmented line of width 5 was drawn through the longitudinal axis of the cells and subsequently measured. Analysis and demographs were created in R.

### Reaction-diffusion equations.

The setup of our mathematical model is based on previous approaches for intracellular protein dynamics (32, 34, 36, 83). Specifically, we present a minimal model to account for DivIVA-mediated MinD localization. The model includes the following set of biochemical reactions: (i) attachment of MinD-ATP (with volume concentration $u_{\mathrm{DT}}$) from the bulk to the membrane with constant rate $k_{D}$; (ii) recruitment of bulk MinD-ATP to the membrane by membrane-bound MinD (with areal concentration $u_{d}$) with rate ${\tilde{k}}_{\mathrm{dD}}$; (iii) hydrolysis and detachment of membrane-bound MinD into bulk MinD-ADP ( $u_{\mathrm{DD}}$) with rate ${\tilde{k}}_{H}$; (iv) reactivation of bulk MinD-ADP by nucleotide exchange to MinD-ATP with rate λ. The system of ensuing reaction-diffusion equations reads as follows:

(4a) $$\partial_{t}u_{\mathrm{DD}}=D_{D}\nabla_{c}^{2}u_{\mathrm{DD}}\;-\;\lambda u_{\mathrm{DD}}$$

(4b) $$\partial_{t}u_{\mathrm{DT}}=D_{D}\nabla_{c}^{2}u_{\mathrm{DT}}\;+\;\lambda u_{\mathrm{DD}}$$

(4c) $$\partial_{t}u_{d}=D_{d}\nabla_{m}^{2}u_{d}\;+\left(k_{D}\;+\;{\tilde{k}}_{\mathrm{dD}}u_{d}\right)u_{\mathrm{DT}}\;-\;{\tilde{k}}_{H}u_{d}$$

where the subscript c or m denotes that the nabla operator acts in the bulk or on the membrane, respectively. These equations are coupled through nonlinear reactive boundary conditions at the membrane surface, stating that the biochemical reactions involving both membrane-bound and bulk proteins equal the diffusive flux onto and off the membrane:

(5a) $$D_{D}\nabla_{n}u_{\mathrm{DD}}{|}_{m}=\;{\tilde{k}}_{H}u_{d}$$

(5b) $$D_{D}\nabla_{n}u_{\mathrm{DT}}{|}_{m}=\;-(k_{D}+{\tilde{k}}_{\mathrm{dD}}u_{d})u_{\mathrm{DT}}$$

Here, the subscript n denotes that we take the nabla operator acting along the outward normal vector of the boundary (membrane). The set of reaction-diffusion equations conserve the total mass of MinD. Hence, the total particle number, $N_{D}$, of MinD obeys the relation

(6) $$N_{D}=\underset{\text{Ω}}{\int }(u_{\mathrm{DD}}\;+\;u_{\mathrm{DT}})\;\mathrm{dV}\;+\underset{\partial \text{Ω}}{\int }u_{d}dS$$

We simulated the set of reaction-diffusion equations in a spherocylindrical geometry in three-dimensional space (3D) using the finite-element software COMSOL v5.4a; for an illustration of the geometry used, see Fig. S7. The length (*L*) and height (*h*) were set to typical values known for B. subtilis cells, L=2.8 μm and h=0.85 μm, respectively. The mean total density of MinD was set to $\left[\text{MinD}\right]=2,450{\text{ μm}}^{-3}\;$ for all simulations (Table S1). We assume that in addition to MinD self-recruitment, MinJ recruits MinD-ATP from the bulk to the membrane and that membrane-bound MinD is stabilized by DivIVA-MinJ complexes on the membrane. We model the interaction of MinD with MinJ and DivIVA implicitly through space-dependent recruitment and detachment rates. To this end, we assume that the recruitment rate is amplified by a factor *α* and that the detachment rate is reduced by a factor *β* at regions of high negative curvature (such as the poles or the septum). This assumption is motivated by experiments which suggest that MinD localization is dependent on MinJ and that DivIVA acts as a scaffold that stabilizes MinJ and MinD (see Discussion). We therefore set the recruitment and detachment rates to $\;k_{\mathrm{dD}}=\alpha {\tilde{k}}_{\mathrm{dD}}\;$ and $k_{H}={\tilde{k}}_{H}/\beta$ at regions of high negative curvature (Fig. S7), where *α* and *β* denote dimensionless amplification and reduction prefactors, respectively. The parameters ${\tilde{k}}_{\mathrm{dD}}$ and ${\tilde{k}}_{H}$ denote the uniform recruitment and detachment rates that one would obtain if interactions between MinD and DivIVA-MinJ complexes were neglected, i.e., if α=β=1 (see below).

### Simulation of the model: polar localization.

In a cell with no preexisting division apparatus, the Min system localizes at the poles of the bacteria (see Discussion). We model this case by setting  α=4 and β=3 at the polar caps and α=β=1 for the remaining part of the rod-shaped geometry (Fig. S7b). The uniform rates were set to ${\tilde{k}}_{\mathrm{dD}}=0.04{\text{ μm}}^{2}\;/\text{ s}$ and ${\tilde{k}}_{H}=0.1{\text{ μm}}^{2}\;/\text{ s}$ as given above. Simulations show that MinD can be pinned to the cell poles for nonuniform kinetic parameters (Fig. 3c, left).

### Depletion of MinD at the poles.

Next, we tested if the polar distribution of MinD decays to a homogeneous protein distribution along the membrane when the rates are uniform over the whole cell body. To this end, we used the steady-state polar distribution of MinD (as obtained above) as the initial condition for a simulation with uniform rates in the entire geometry, i.e., α=1, β=1. We found that for uniform rates, MinD proteins preferentially localize near the cell center (Fig. 3c, left to right). The reason for this unexpected inhomogeneous protein distribution is a purely geometric effect (see Discussion).

### Localization at septum.

The curvature-sensing protein DivIVA targets the division site and guides MinJ and MinD to the septum (see Discussion). Above, we showed that MinD localizes to the cell poles if the recruitment and detachment rate of MinD are altered at the poles due to interactions with MinJ and DivIVA. For uniform rates, however, the MinD density distribution is spread around midcell but not sharply localized at the septum as observed in experiments. Sharp localization of MinD at midcell requires interaction with DivIVA and MinJ, and we therefore model this case in the same way as for polar localization. First, we define a narrow region with width $s_{w}=0.14\text{ μm}$ at midcell, which represents the septum (Fig. S7c). We set again α=4 and β=3 at this geometric region to model the interactions of MinD with MinJ and DivIVA implicitly through a modified recruitment and detachment rate. Simulations of the model show that MinD localizes sharply at the septum (Fig. 3d, left to right).

### Parameter dependence of the simulation results.

Since we consider steady-state solutions of the reaction-diffusion system in equations 4 and 5, our qualitative results are not sensitive against variations of the kinetic parameters (Table S1). Changing the values of the kinetic parameters would only shift the dynamic equilibrium state, without affecting the protein distributions qualitatively. There is only one exception, which is the nucleotide exchange rate, *λ*, or, more precisely, the reactivation length scale $l=\sqrt{D_{D}/\lambda }$.

Since nucleotide exchange and diffusion are the main reasons for the geometric effect discussed above, the qualitative steady-state density distributions may depend on l. We will discuss two relevant limits which affect the redistribution of MinD to midcell. (i) Let us assume that the reactivation of detached MinD-ADP to MinD-ATP is instantaneous and hence λ is very large. In this case, the reactivation length would be much smaller than the radius of curvature at the poles R, i.e., l≪R. A very small value of l implies that detached proteins can rebind the membrane without delay. Therefore, in this case, there is no geometric effect and the steady-state density distribution of MinD would be homogeneous. (ii) Next, let us assume that λ is very small, such that the reactivation length becomes much larger than the length of the bacteria L, i.e., l≫L. This would imply that proteins detaching from the membrane diffuse a long distance until they exchange their nucleotide and become able to rebind the membrane again. In this case, the MinD density distribution would be also homogeneous. However, due to the small value of λ, inactive MinD-ADP proteins are abundant in the cytosol and only few MinD-ATP proteins attach to the membrane, resulting in low membrane densities.

The geometric effect (see above) is present if the value of l lies between the radius of curvature at the poles and the length of the bacteria, i.e., R<l<L. Therefore, our qualitative results are not sensitive to the exact choice of l as long as the inequality above is fulfilled. For our parameters, we have R≈0.42  μm,  *L* =2.8 μm, and $l=\sqrt{D_{D}/\text{λ}}\approx 1.6\text{ μm}$. In summary, this geometric effect is quite robust and does not require the fine-tuning of parameters. For an in-depth discussion of the geometric effect and its dependence on various system parameters, see reference 83.

### PALM and cluster analysis.

Photoactivated localization microscopy (PALM) imaging was performed with the microscope system ELYRA P.1 (Zeiss) and the accompanying Zen software. It is equipped with a 405-nm diode-laser (50 mW), a 488-nm laser (200 mW), a 561-nm laser (200 mW), and a 640-nm laser (150 mW). Furthermore, an alpha Plan-Apochromat 100×/1.46 oil differential inference contrast (DIC) M27 objective (Zeiss) was used, in combination with a 1.6× Optovar. The filter sets were the following: a 77 HE GFP+mRFP+Alexa 633 shift-free (EX TBP 483 + 564 + 642, BS TFT 506 + 582 + 659, EM TBP 526 + 601 + 688), a 49 DAPI shift-free (EX G 365, BS FT 395, EM BP 445/50), a BP 420–480/LP 750, a BP 495–550/LP 750, an LP 570, and an LP 655 filter set. Images were recorded with an Andor EMCCD camera iXon DU 897. Samples expressing mNeonGreen were illuminated with the 488-nm laser at 7.4 mW. Samples expressing Dendra2 or PAmCherry were illuminated with an excitation laser (561 nm, 5.3 mW) and an activation laser (405 nm). To avoid cooccurrence of multiple events in the same spot, the power of the activation laser was increased stepwise from 0.008 mW to 1.6 mW. MinJ-mNeonGreen was illuminated in pseudo-TIRF (total internal reflection fluorescence) mode and recorded at 20 Hz with 200 camera gain, while Dendra2-MinD and DivIVA-PAmCherry were imaged with the same camera settings in regular wide field. Analysis was performed in the Zen Black (Zeiss) software. Detection of single emitters was performed with a peak mask size of 9 pixels and a minimum peak intensity-to-noise ratio of 6.0; overlapping emitters were discarded. Localization was extrapolated via a 2D Gaussian fitting, and images were drift corrected utilizing a fiducial-based mode with at least 3 beads in focus. Filtering was used to minimize noise, background, and out-of-focus emitters and to exclude beads from the evaluation, according to Table 7, which were different for each respective fluorophore.

**TABLE 7:** Filter parameters for PALM imaging of the different strains^*a*^

Strain or FP	point spread function (PSF) at half maximum [nm]	No. of photons
Dendra2-MinD	70–160	70–250
MinJ-mNeonGreen	70–160	70–300
DivIVA-PAmCherry	60–170	50–500

a Filters were chosen according to the fluorophore (FP) behavior in PALM to eliminate background and signal of fluorescent beads from the results.

Cluster analysis was performed in R 3.3.1 (93) utilizing the DBSCAN package (96, 97) including OPTICS (98). Clusters were determined by applying the OPTICS algorithm to the respective molecule tables generated via PALM. The minimal number of points that define a cluster (minPts) was defined as 10, reflecting apparent clusters seen in rendered PALM imaging, and a minimum distance between cluster edge points (epsCl) of 20 and 30 nm for MinD and DivIVA, respectively, according to the observed density of protein localization.
