# Supplementary figures and images for: Dynamics of the Bacillus subtilis Min System
Source: mBio. 2021 Apr 13;12(2):e00296-21. doi: 10.1128/mBio.00296-21 (PMC8092234; doi:10.1128/mBio.00296-21)

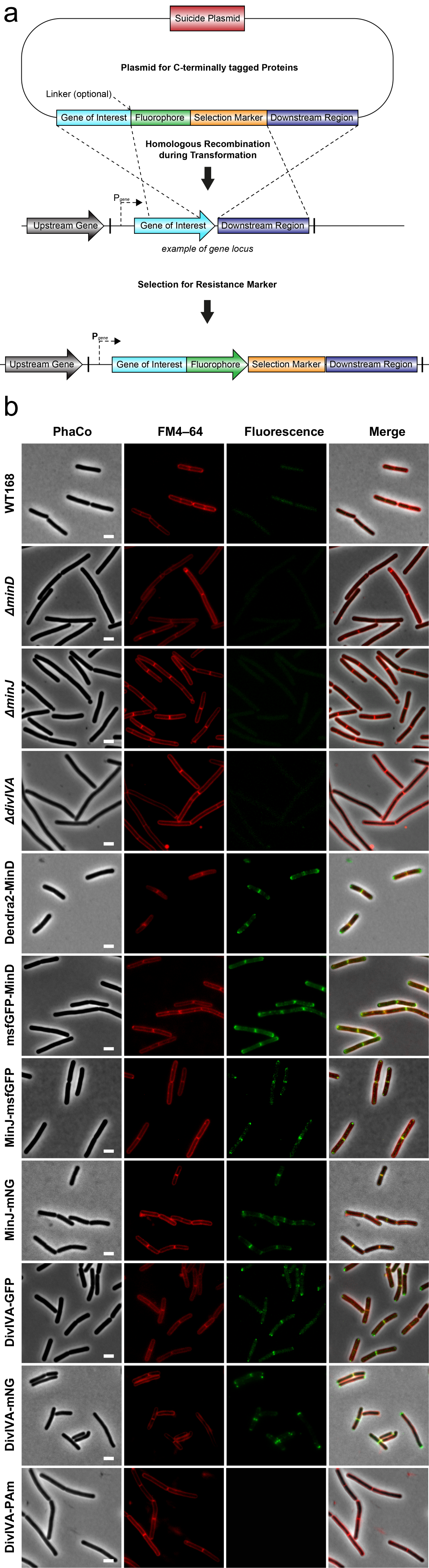

Supplement: FIG S1 [file mBio.00296-21-sf001.tif]

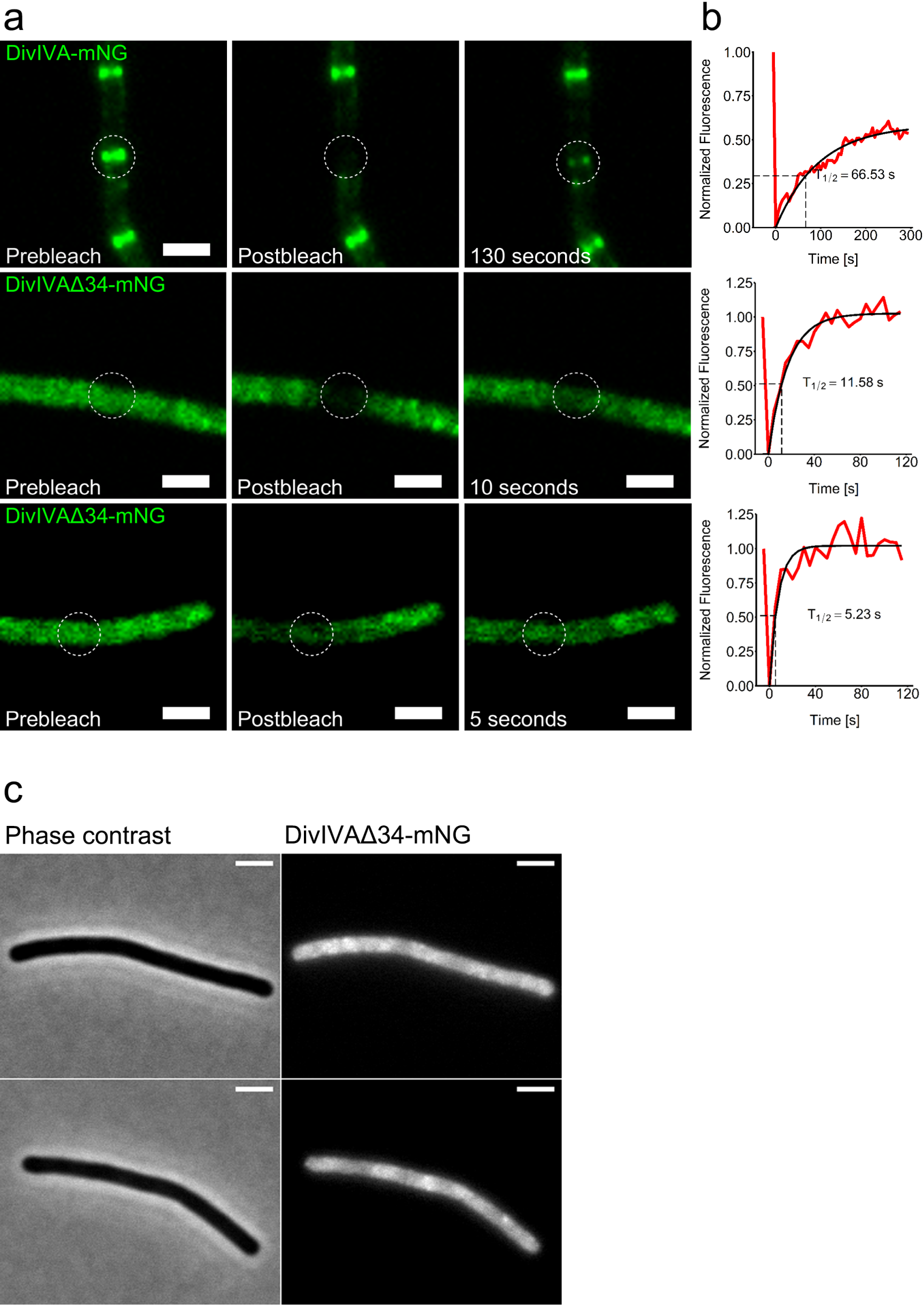

Supplement: FIG S2 [file mBio.00296-21-sf002.tif]

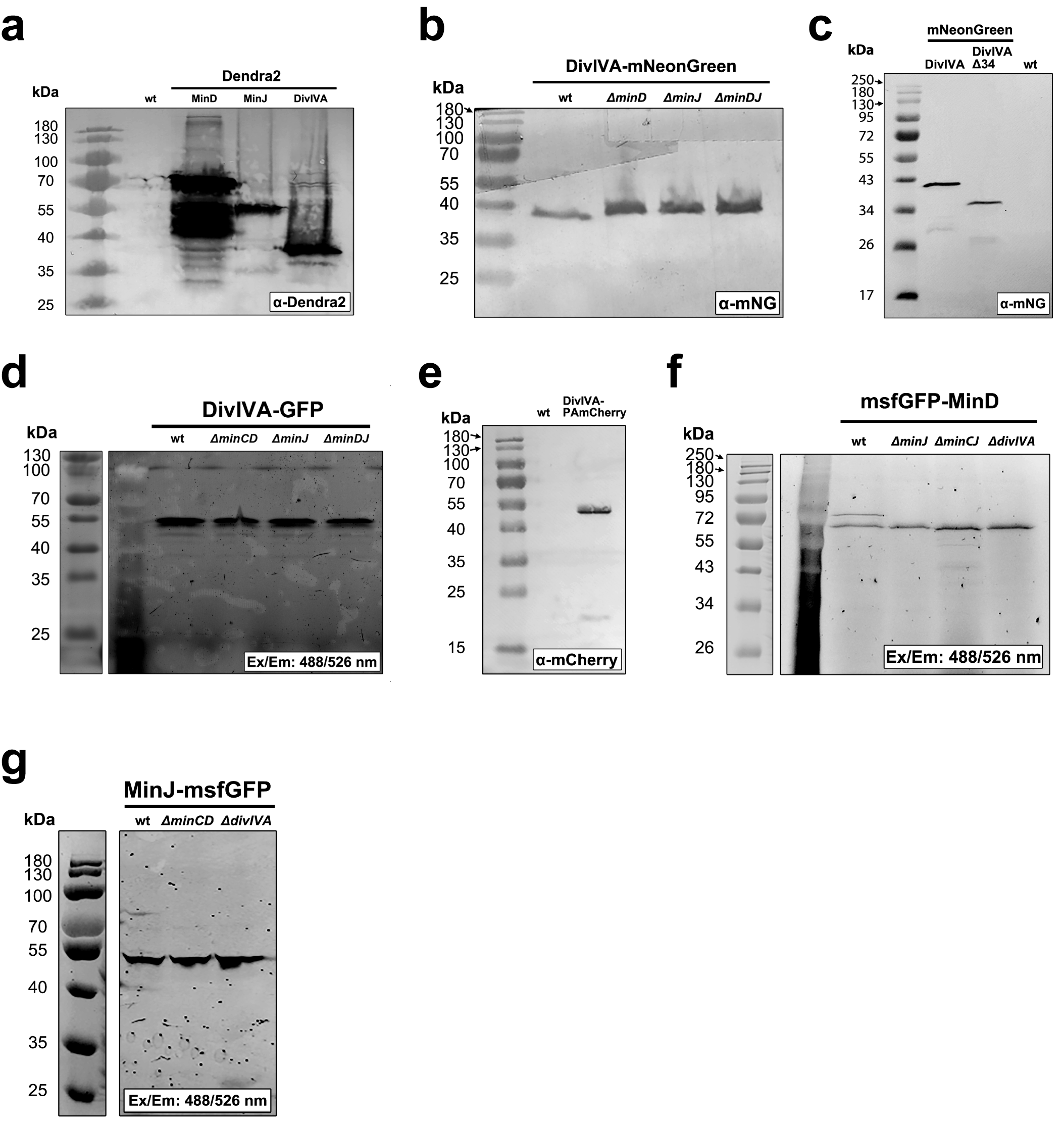

Supplement: FIG S3 [file mBio.00296-21-sf003.tif]

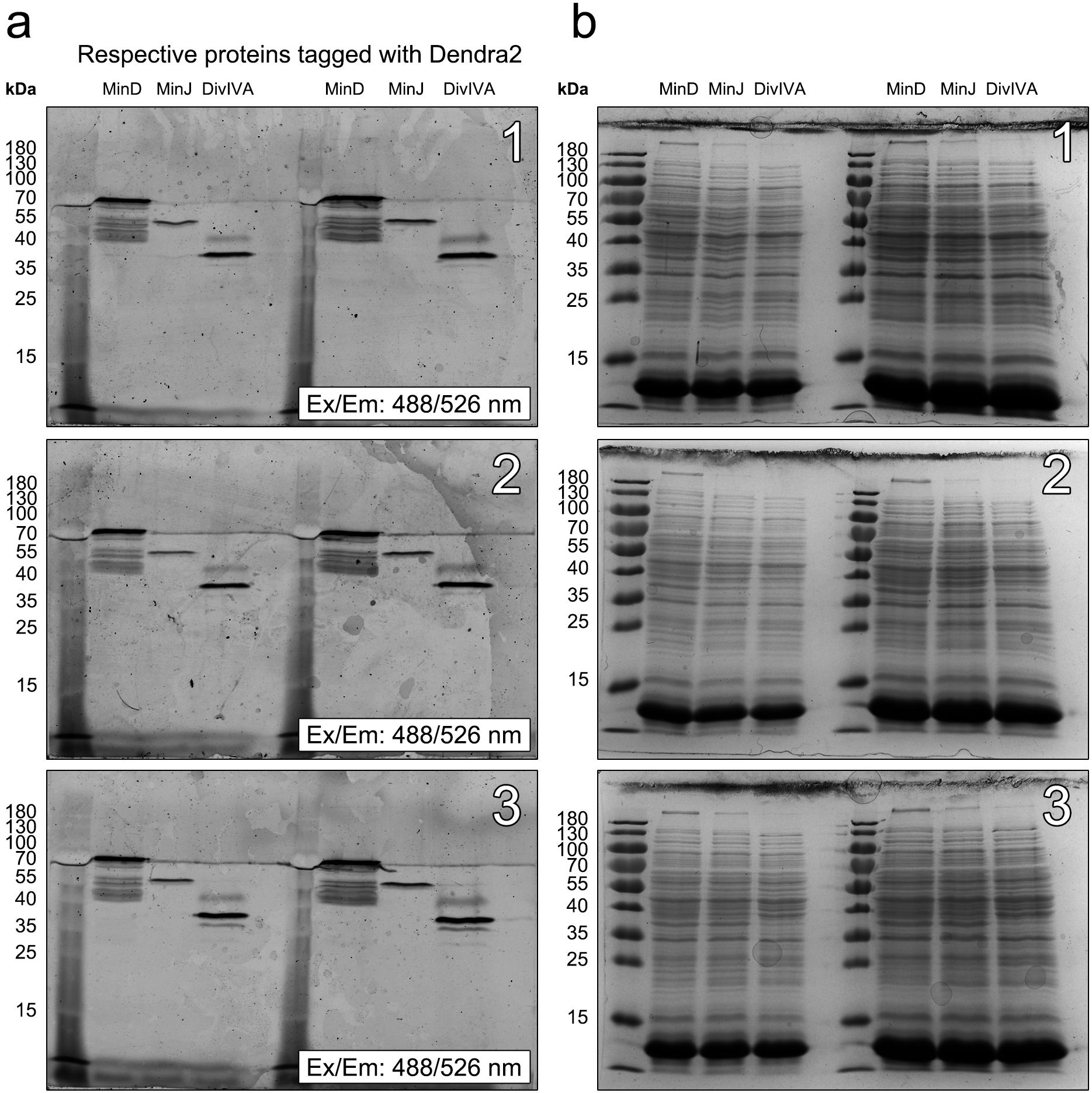

Supplement: FIG S4 [file mBio.00296-21-sf004.tif]

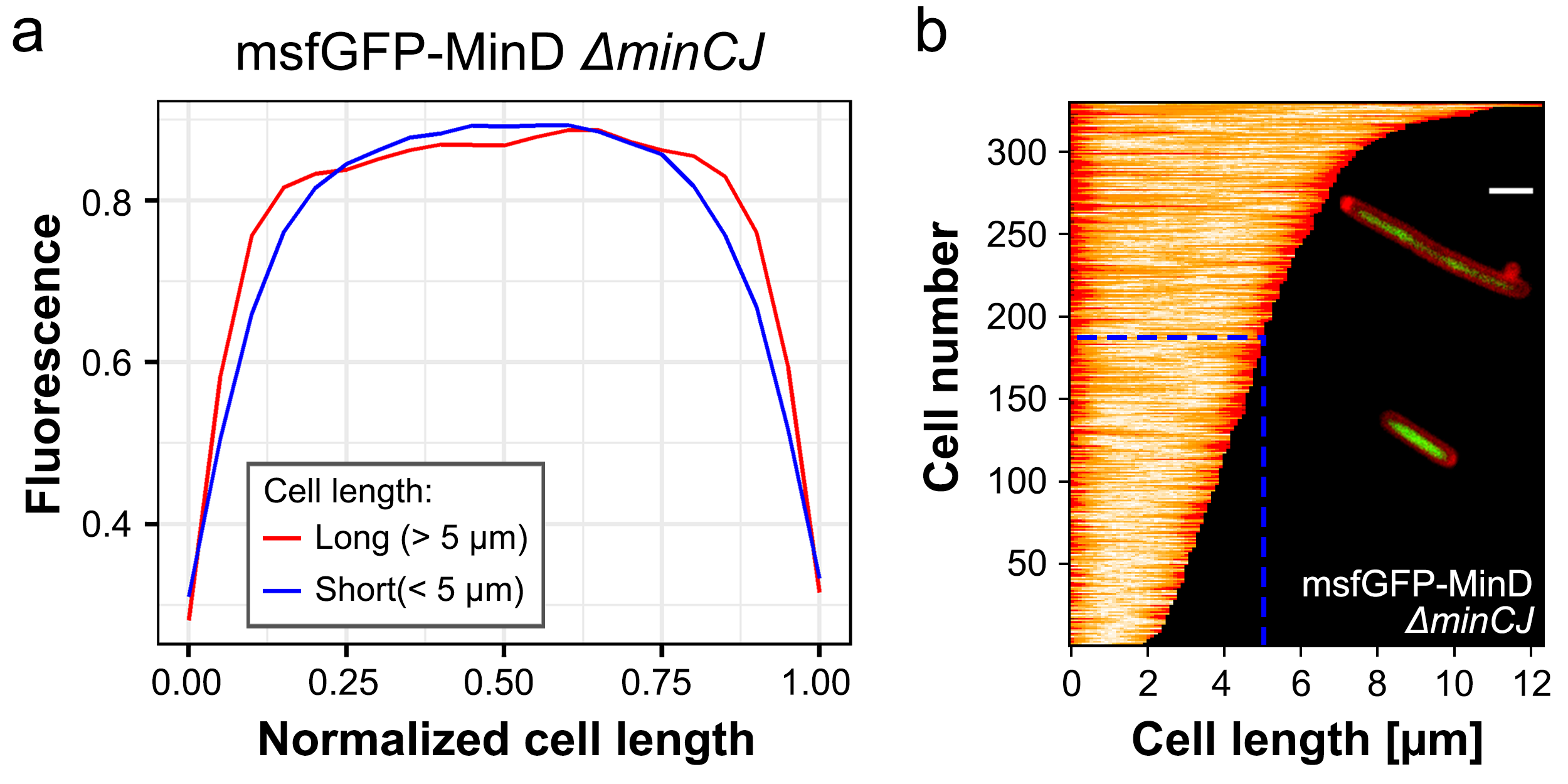

Supplement: FIG S5 [file mBio.00296-21-sf005.tif]

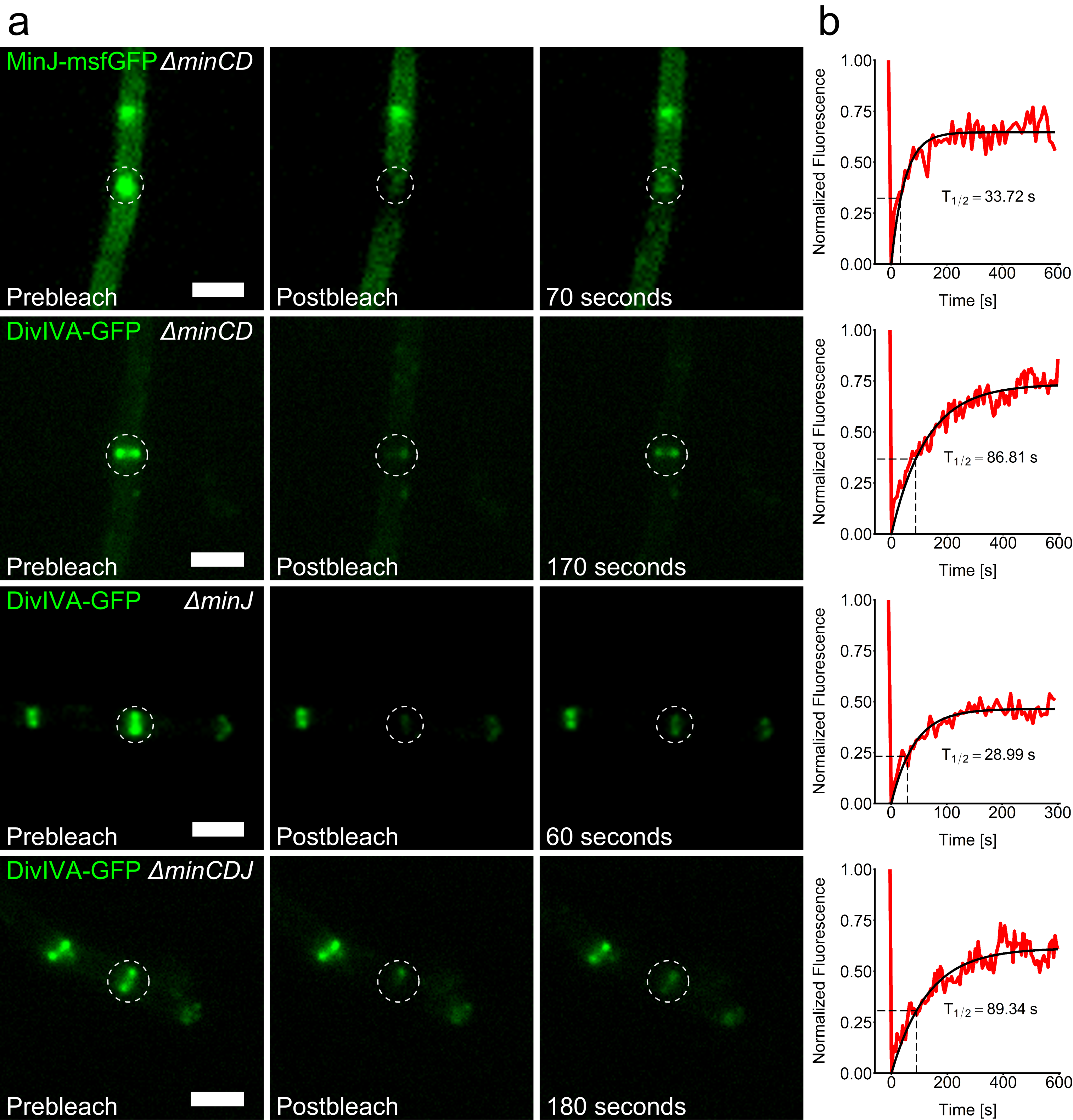

Supplement: FIG S6 [file mBio.00296-21-sf006.tif]

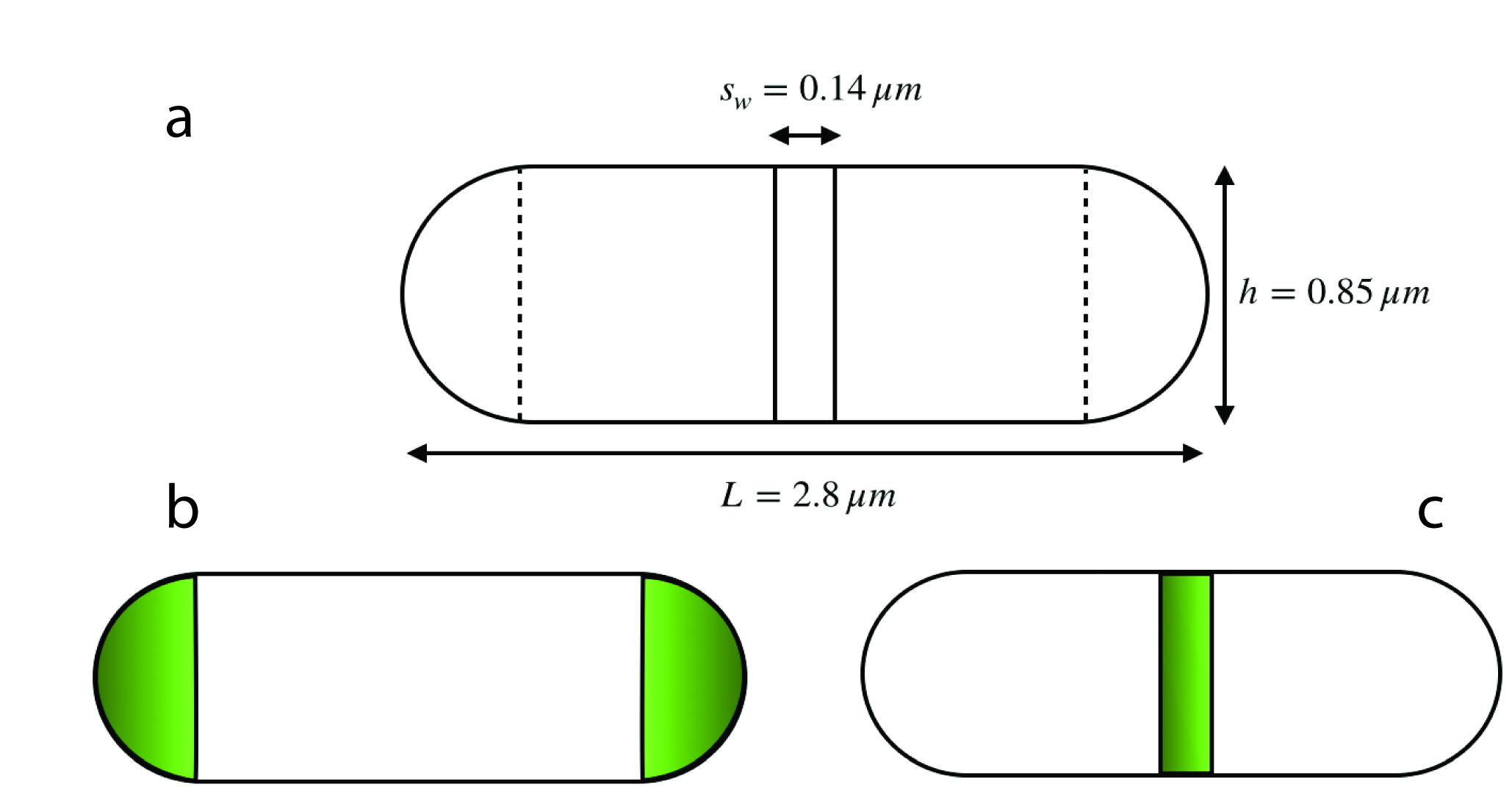

Supplement: FIG S7 [file mBio.00296-21-sf007.tif]

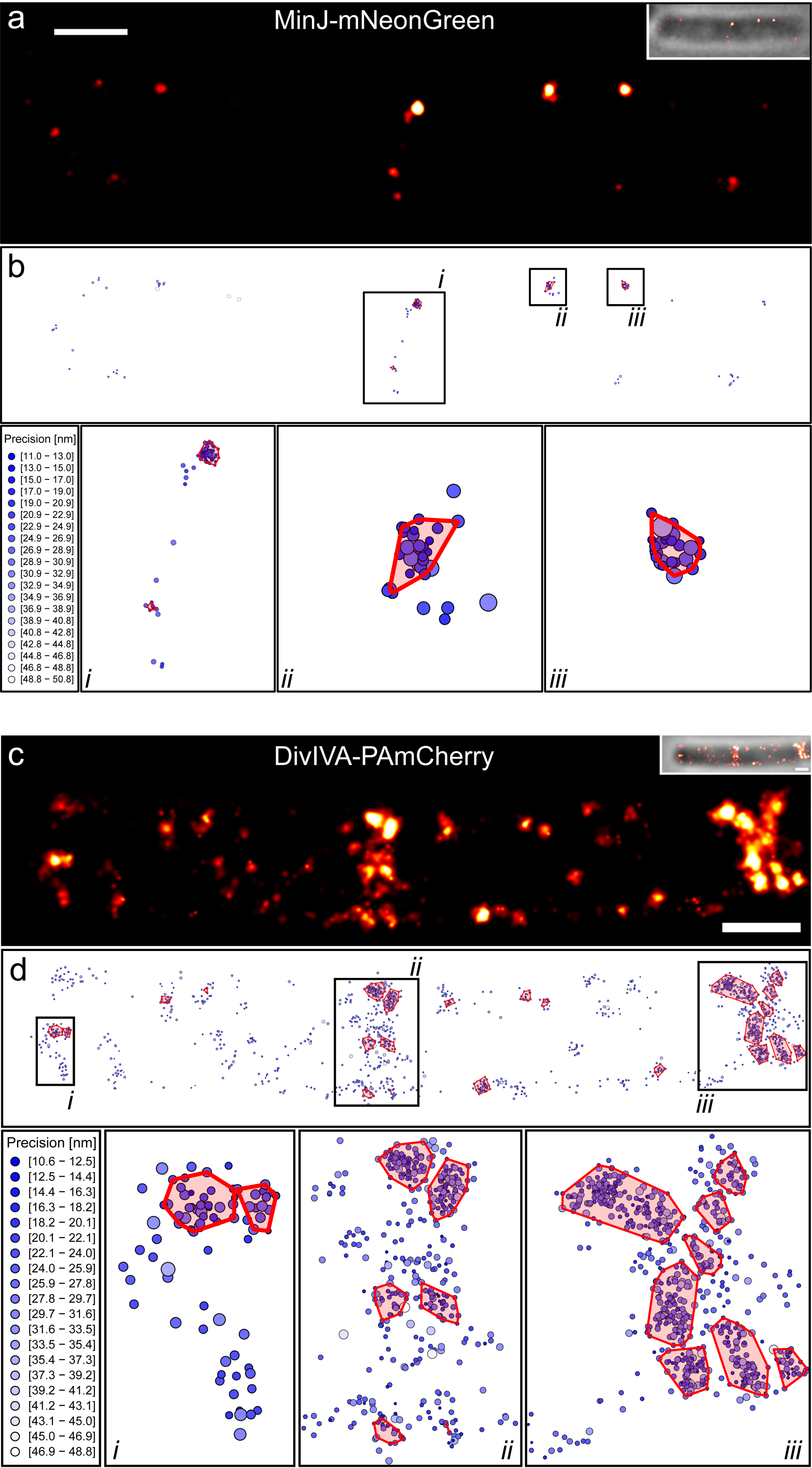

Supplement: FIG S8 [file mBio.00296-21-sf008.tif]
