# Supplementary material for: Dynamics of the Bacillus subtilis Min System
Source: mBio. 2021 Apr 13;12(2):e00296-21. doi: 10.1128/mBio.00296-21 (PMC8092234; doi:10.1128/mBio.00296-21)
Supplement: TABLE S1 [file mBio.00296-21-st001.docx]

## Supplementary Tableb S1

| Parameter | Symbol | Value |
| --- | --- | --- |
| Bulk Diffusion | $D_{D}$ | $16 \mu m^{2}\cdot s^{-1}$ |
| Membrane Diffusion | $D_{d}$ | $0.06 \mu m^{2}\cdot s^{-1}$ |
| Mean total density | $\left[ MinD \right]$ | $2450 \mu m^{-3}$ |
| Attachment rate | $k_{D}$ | $0.068 \mu m\cdot s^{-1}$ |
| Uniform recruitment rate | $\tilde{k}_{dD}$ | $0.04 \mu^{2}\cdot s^{-1}$ |
| Uniform hydrolysis rate | $\tilde{k}_{H}$ | ${0.1 s}^{-1}$ |
| Recruitment rate amplification factor | $\alpha$ | $4$ |
| Hydrolysis rate reduction factor | $\beta$ | $3$ |
| Nucleotide exchange rate | $\lambda$ | ${6 s}^{-1}$ |
| Cell length | $L$ | $2.8 \mu m$ |
| Cell width | $h$ | $0.85 \mu m$ |
